# Immune response of healthy horses to DNA constructs formulated with a cationic lipid transfection reagent

**DOI:** 10.1186/s12917-015-0452-3

**Published:** 2015-06-23

**Authors:** Christiane L. Schnabel, P. Steinig, M. Koy, H.-J. Schuberth, C. Juhls, D. Oswald, B. Wittig, S. Willenbrock, H. Murua Escobar, C. Pfarrer, B. Wagner, P. Jaehnig, A. Moritz, K. Feige, J.-M. V. Cavalleri

**Affiliations:** University of Veterinary Medicine Hannover, Clinic for Horses, Buenteweg 9, 30559 Hannover, Germany; University of Veterinary Medicine Hannover, Immunology Unit, Bischofsholer Damm 15, 30173 Hannover, Germany; Mologen AG, Fabeckstrasse 30, 14195 Berlin, Germany; Foundation Institute Molecular Biology and Bioinformatics, Freie Universitaet Berlin, Berlin, Germany; University of Veterinary Medicine Hannover, Small Animal Clinic, Buenteweg 9, 30559 Hannover, Germany; Division of Medicine, Clinic III, Haematology, Oncology and Palliative Medicine, University of Rostock, 18057 Rostock, Germany; University of Veterinary Medicine Hannover, Institute of Anatomy, Bischofsholer Damm 15, 30173 Hannover, Germany; Department of Population Medicine and Diagnostic Sciences, College of Veterinary Medicine, Cornell Universit, 240 Farrier Rd, Ithaca, NY 14853 USA; pj statistics, Niedstrasse 16, 12159 Berlin, Germany; Department of Veterinary Medicine, Clinical Sciences, Clinical Pathology and Clinical Pathophysiology, Justus-Liebig-Universitaet, Frankfurter Strasse 126, 35392 Giessen, Germany

**Keywords:** Equine melanoma, Grey horse, MIDGE vector, Cytokines, CpG, IL-12, IL-18, Transfection reagent, DNA vaccine, Cationic lipid

## Abstract

**Background:**

Deoxyribonucleic acid (DNA) vaccines are used for experimental immunotherapy of equine melanoma. The injection of complexed linear DNA encoding interleukin (IL)-12/IL-18 induced partial tumour remission in a clinical study including 27 grey horses. To date, the detailed mechanism of the anti-tumour effect of this treatment is unknown.

**Results:**

In the present study, the clinical and cellular responses of 24 healthy horses were monitored over 72 h after simultaneous intradermal and intramuscular application of equine IL-12/IL-18 DNA (complexed with a transfection reagent) or comparative substances (transfection reagent only, nonsense DNA, nonsense DNA depleted of CG).

Although the strongest effect was observed in horses treated with expressing DNA, horses in all groups treated with DNA showed systemic responses. In these horses treated with DNA, rectal temperatures were elevated after treatment and serum amyloid A increased. Total leukocyte and neutrophil counts increased, while lymphocyte numbers decreased. The secretion of tumour necrosis factor alpha (TNFα) and interferon gamma (IFNγ) from peripheral mononuclear blood cells *ex vivo* increased after treatments with DNA, while IL-10 secretion decreased. Horses treated with DNA had significantly higher myeloid cell numbers and chemokine (C-X-C motif) ligand (CXCL)-10 expression in skin samples at the intradermal injection sites compared to horses treated with transfection reagent only, suggesting an inflammatory response to DNA treatment.

In horses treated with expressing DNA, however, local CXCL-10 expression was highest and immunohistochemistry revealed more intradermal IL-12-positive cells when compared to the other treatment groups.

In contrast to non-grey horses, grey horses showed fewer effects of DNA treatments on blood lymphocyte counts, TNFα secretion and myeloid cell infiltration in the dermis.

**Conclusion:**

Treatment with complexed linear DNA constructs induced an inflammatory response independent of the coding sequence and of CG motif content. Expressing IL-12/IL-18 DNA locally induces expression of the downstream mediator CXCL-10.

The grey horses included appeared to display an attenuated immune response to DNA treatment, although grey horses bearing melanoma responded to this treatment with moderate tumour remission in a preceding study. Whether the different immunological reactivity compared to other horses may contributes to the melanoma susceptibility of grey horses remains to be elucidated.

**Electronic supplementary material:**

The online version of this article (doi:10.1186/s12917-015-0452-3) contains supplementary material, which is available to authorized users.

## Background

Melanoma is one of the most common equine cutaneous neoplasms [1–3]. Prevalence is up to 80 % in grey horses older than 15 years, while horses of other colours are rarely affected by this disease [4, 5]. To date, there is no satisfactory therapeutic approach available in advanced cases of grey horse melanoma [1, 4, 6]. One experimental *in vivo* approach is immunotherapy with melanoma antigens and/or immune modulating cytokines encoded by deoxyribonucleic acid (DNA) vectors taking advantage of systemic effects on metastases [7–9]. Interleukin (IL)-12 and IL-18 have been employed as they synergistically stimulate cytotoxic T-cells and natural killer cells [10–12], which are typically involved in anti-tumour immune responses. The two cytokines improve antigen presentation [13] and form a link between innate and adoptive immunity [14, 15]. Furthermore, anti-angiogenic properties have been described which could contribute to anti-tumour effects [16, 17].

A moderate decrease in the tumour volume of melanomas was observed by Mählmann et al. [18] during a study period of 120 days after immunotherapy of 27 grey horses with mixed linear DNA vectors (minimalistic immunologic defined gene expression (MIDGE)-Th1) encoding equine IL-12 and IL-18. These DNA vectors were formulated with the transfection reagent SAINT-18 and administered simultaneously intramuscularly (i.m.) and peritumourally intradermally (i.d.).

MIDGE-Th1 vectors are minimalistic linear double-stranded DNA molecules lacking plasmid backbone sequences. The vectors are covalently closed with single-strand hairpin loops at both ends. They contain the expression cassette only, i.e. a promoter, the coding sequence of interest and a polyadenylation site [19, 20]. One of the ends is covalently bound to a nuclear localisation signal peptide, triggering an improved humoral and cellular response of a T_H_1 phenotype [21, 22]. *In vivo* transfection is established and can be facilitated by the formulation of the DNA vectors with cationic lipids, such as SAINT-18, to form complexes with these molecules [23, 24].

Signs of a systemic immune response, i.e. the development of fever 12 h after administration of MIDGE-Th1 formulated with SAINT-18 and the size reduction of metastases which had not been treated locally, were observed in the study of Mählmann et al. [18]. Local immunological responses were indicated by signs of acute inflammation (swelling, reddening, etc.) and depigmentation of the skin at the site of injection. The exact mechanisms of this anti-tumour therapy are still unknown.

Effects of DNA vectors are usually explained by their transgene products [9, 19, 23, 25], by immunologic effects caused by CG motifs randomly contained in the vector constructs which activate toll-like receptor (TLR-) 9 pathways [26–28], by reactions triggered by interaction with cytosolic receptors for dsDNA [29–33], or by combinations of these effects.

The mechanisms of the immunotherapeutic effects of MIDGE-Th1 encoding equine IL-12 and IL-18 demonstrated by Mählmann et al. [18] in grey horses affected with melanoma need to be elucidated in order to improve the directed clinical use of anti-tumour treatments. Therefore, the aim of this study was the characterisation of the immune response to the treatment by investigation of various candidate immune parameters on the systemic and local level in healthy horses. Furthermore, potential immunologically active components of the therapeutic were analysed for their contribution to the immune response. This was conducted comparatively in four treatment groups, representing effects by transfection reagent only, transgene products, CG motifs or DNA independent of its sequence.

## Methods

### Ethical statement

All procedures were carried out according to the ethical guidelines of the law on animal welfare (Tierschutzgesetz) approved by the “Niedersächsisches Landesamt für Verbraucherschutz und Lebensmittelsicherheit” in the animal experiment No. 33.9-42502-04-11/0399. Informed consent was obtained from all animal owners.

### Study design

A prospective, randomised, double-blinded study was performed in 24 horses assigned randomly to four groups of six horses each (A–D) by the criteria of colour, age and breed (Table 1).

**Table 1:** Distributions of horses within treatment groups

Treatment group	Treatment	Mean age (years)	Mean weight (kg)	Mares (*n*)	Geldings (*n*)	Stallions (*n*)	WBl (*n*)	ThB (*n*)	Grey (*n*)	Non-grey (*n*)
A	SAINT18	11.1	561	2	3	1	4	2	2	4
B	SAINT18+ eqIL12 + eqIL18	10.8	589	2	3	1	5	1	2	4
C	SAINT18+ eqIL12-ATG + eqIL18-ATG	10.1	548	0	5	1	4	2	2	4
D	SAINT18+ eqIL12-ATG-CG + eqIL18-ATG-CG	11.3	591	2	4	0	5	1	2	4

*IL* interleukin, *−ATG* nonsense DNA, *−ATC-CG* nonsense DNA depleted of CG, *WBl* warmblood, *ThB* thoroughbred

### Animals

Clinically healthy horses (6 mares, 15 geldings and 3 stallions) aged between 2 and 21 years (mean 10.6 years) with a body weight of 425–680 kg (mean 572 kg) were included in the study. There were six Thoroughbred (ThB) type horses (two Arabians, two Arabian-mix and two English ThBs) and 18 Warmblood (WBl) type horses (nine Hanoverian WBls, one Hessian WBl, one Polish WBl, two Pura Raza Españolas (PRE), one PRE-mix, one Standardbred, one Oldenburg WBl, and one Westfalian WBl; Table 1). One horse of treatment group A had a history of insect bite hypersensitivity and one horse of each of the treatment groups A, B and C had a history of chronic obstructive bronchitis/bronchiolitis, but all horses were asymptomatic throughout the study period. No systemic and potentially immunomodifying treatments were administered in the two weeks preceding the experiments.

The animals were kept in stables of the Clinic for Horses, University of Veterinary Medicine, Hannover, Foundation, housed in standard single boxes on straw or wood shavings under a natural light–dark cycle of German summer (15–16.3 h of light, sunrise between 05:12 and 05:44, light from large windows or adjacent paddocks). The horses were fed hay and concentrates twice a day (at 07:00 and 18:00), according to their body weight, and had access to water ad libitum. Horses had access to an outdoor 20 × 60 m sand area once a day, in the late afternoon or early evening, with a minimum of 2 h rest prior to examination and sampling.

**Fig. 1:**
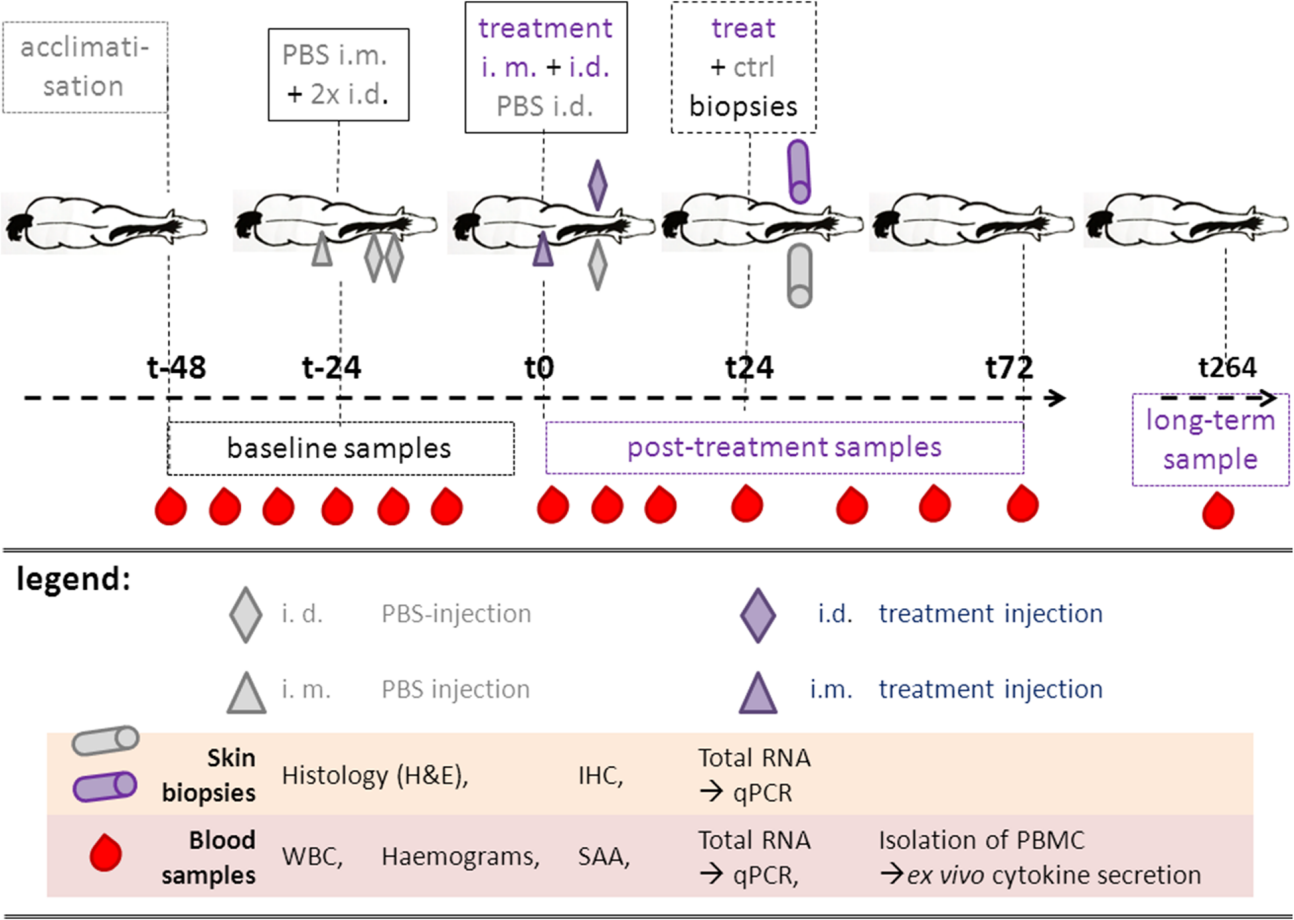
Procedure Test procedure illustrated in course of time. The horses were left to acclimatise for at least three days. At t-24, the horses were injected with PBS (grey symbols) i.m. (0.5 ml) and i.d. (2 × 0.5 ml). Baseline samples (blood) as internal controls for systemic parameters were acquired from t-48 – t0. At t0, horses were injected with treatments A – D (purple symbols) i.m. (0.5 ml) and i.d. (0.5 ml) and contralaterally with PBS (grey symbol, 0.5 ml). From t6 – t72, post-treatment samples (blood) of systemic parameters were acquired. At t24, skin biopsies were acquired of treatment (purple) and PBS control (grey) sites. Eleven days post-treatment (t264), one long-term sample was acquired at the home stable of each horse. General examinations were performed at all sampling times. Blood samples were used to determine WBC, haemograms, SAA, cytokine mRNA and *ex vivo* cytokine secretion by PBMC. Skin samples were used to perform histological examinations, IHC of IL-12, IL-18 and calprotectin, and qPCR of cytokine and chemokine mRNA

The horses were allowed to acclimatise for at least 2 days before starting the experiment and were trained by classical and operant conditioning for blood sampling to minimise stress during blood collection. Horses stayed in the clinic for 8 days (t-96 – t72). Examinations were performed and samples were acquired at the horses’ home stables at day 11 (t264) after treatment (Fig. 1).

### Test items and groups

Four horses at a time were kept in the clinic and each was treated with one of the four different treatments, A–D. Injection solutions were provided in blinded tubes in a volume of 1 ml buffered with phosphate-buffered saline (PBS). Treatment letter codes were kept at the DNA vector manufacturing site and opened only after all study data had been acquired and evaluated.

Group A received only the transfection reagent (SAINT-18, Synvolux Therapeutics B.V., Groningen, Netherlands; containing 6 nmol/ml 1-methyl-4-(cis-9-dioleyl) methyl-pyridinium-chloride).

Group B received SAINT-18 and linear DNA vectors (MIDGE-Th1, eqIL12 and IL-1beta receptor antagonist protein (ILRAP)-eqIL18) encoding equine IL-12 [NM_001082511.1; NM_001082516.1] and IL-18 [NM_001082512.1] fused to the coding sequence of the IL-1beta receptor antagonist protein (ILRAP) signal peptide.

Group C received SAINT-18 and non-expressing MIDGE-Th1 vector of equine IL-18 with point mutations in all ATG sequences to delete potential start codons (nonsense DNA; eqIL18-ATG). These vectors had 1.02 times the number of total CG sequences, thus, potential CG motifs, as those given in the dose of group B.

Group D received SAINT-18 and nonsense DNA, similar to group C, but with inverted CG motifs (eqIL18-ATG-CG).

### DNA vectors

The construction and synthesis of MIDGE-Th1 vectors was performed by MOLOGEN AG (Berlin, Germany), analogous to the method described by Schirmbeck et al. [21]. Briefly, the expression cassettes containing the cytomegalo virus immediate-early enhancer/promoter, a chimeric intron, the gene sequence (eqIL12; eqIL18; eqIL18-ATG or eqIL18-ATG-CG) and the SV40 late protein polyadenylation site were inserted into the plasmid pMCV1.4. The coding sequence for the ILRAP signal peptide was fused to the eqIL18 coding sequence in all eqIL18 MIDGE vectors. A digestion with the restriction endonuclease Eco31-I (MBI Fermentas, Vilnius, Lithuania) was performed to set the expression cassette free. The open ends of the expression cassette were sealed with loop-forming oligodeoxyribonucleotides, one of them linked to a peptide nuclear localisation sequence (a peptide with the amino acid sequence PKKKRKVEDPYC) [21]. Unbound DNA fragments were degraded with T7 DNA polymerase (MBI Fermentas, Vilnius, Lithuania) followed by purification of MIDGE-Th1 vectors.

### Formulation of DNA vectors or phosphate-buffered saline with SAINT-18

The transfection reagent SAINT-18 (0.5 ml dissolved in water) was vortexed for 1 min and then mixed manually with 2× PBS (Biochrom AG, Berlin, Germany), or the DNA was dissolved in 2× PBS, obtaining the test item at a volume of 1 ml. Lipid-DNA complexes were allowed to form at room temperature (rt) for approximately 10 min prior to injection, achieving a mixture of 1 ml in 1× PBS. MIDGE-Th1/SAINT-18 complexes in groups B, C and D were formed at a ratio of 1 mg DNA to 0.75 μmol SAINT-18 and the DNA concentration was 0.4 mg/ml 1× PBS.

### Treatment

All time points (t) in the following are reported as h before or after treatment (t-48 to t264; Fig. 1).

The test item dose of 1 ml was split. One half (0.5 ml) was injected into the pectoralis muscle. The other 0.5 ml was injected i.d. into the middle third of the neck under subcutaneous local anaesthesia with 1 ml of lidocaine (Lidocainhydrochlorid 2 %, Bela-Pharm, Vechta, Germany) after aseptic preparation of the i.d. injection site with chlorhexidine (Hibiscrub, tk pharma trade, Hasbergen, Germany). The i.d. injection site (*treat*) was marked.

In order to evaluate local effects related to the application procedure, but unrelated to the test items at the time of treatment (t0), the contralateral side of the neck was treated with 0.5 ml of PBS i.d. (*ctrl*) after the same preparation and local anaesthesia as the *treat* site.

Clipping and shaving of i.d. injection sites was performed at t-36 to allow local irritation to resolve before injections (t0) and biopsy sampling (t24).

Twenty-four hours prior to treatment (t-24), the horses were injected with PBS in the same manner, but contralaterally (0.5 ml i.m. and 1 ml i.d. split into two injections of 0.5 ml each, injected at least 5 cm distant from each other and 20 cm from the injection sites of t0; Fig. 1). This injection of PBS was performed in order to evaluate the systemic effects of the injection procedure unrelated to the test item.

### Examinations

The horses underwent a general clinical examination 4 times a day between t-48 and t48 and twice a day during the rest of their stay.

The skin at the injection sites was also examined clinically at these time points and digital photographs were taken. Changes were described in the categories oedema, pain, heat, depigmentation and exudate by a non-linear scale (0–3 representing absent – high-grade, respectively). Scores were summarised as the sums of all categories in a single clinical skin score.

Blood samples were drawn at 09:00, 15:00 and 21:00 from t-24–t24, and at 09:00 and 21:00 from t-48– −24 and from t24–t72. One general examination, clinical scoring of the injection site and blood sampling were performed on each horse at t264.

### Blood sampling

Blood samples were drawn from the jugular vein after disinfection on the surface area with ethanol, by use of a vacutainer system (Vacuette, greiner bio-one, Frickenhausen, Germany) with a 20 G single-use cannula. Blood was collected in ethylenediaminetetraacetate (EDTA)-coated tubes for routine clinical laboratory measurements, in clot-activator-coated tubes for serum, in heparinised (sodium-heparin) tubes for the determination of SAA, and in PAXgene blood tubes (BD, Heidelberg, Germany) for the isolation of peripheral blood mononuclear cells (PBMC) and for the conservation of total messenger ribonucleic acid (mRNA).

### Serum preparation

Blood was allowed to clot at rt for 2 h and was then centrifuged at 1430 × g for 6 min. Serum without clots was stored in aliquots at 4 °C for a maximum of 48 h until further analysis.

### Routine clinical laboratory measurements

Analysis by routine clinical laboratory measurements included total plasma protein (TPP), haematocrit (Hct), white blood cell counts (WBC) and differential haemograms, measured by a manual refractometer (TPP), a Sysmex kx-21 N (Sysmex Deutschland GmbH, Norderstedt, Germany) (Hct, WBC) and an ADVIA 120 (Siemens Healthcare Diagnostics GmbH, Eschborn, Germany) (haemograms), respectively. Due to the availability of laboratory devices, differential haemograms were obtained after storage of EDTA-blood at rt for a maximum of 36 h. Absolute leukocyte subset (neutrophils, lymphocytes, monocytes) counts were calculated from percentages of the ADVIA measurement and total WBC measured immediately after sampling by Sysmex kx-21 N.

### Serum amyloid A determination

Serum amyloid A was determined by an immunoturbidimetric latex agglutination test (LZ Test Eiken SAA, Eiken Chemical Co. Ltd., Tokyo, Japan). Limits of detection were 5–300 μg/ml. This assay for human serum amyloid A (SAA) had been evaluated for horses [34].

### Isolation of peripheral blood mononuclear cells

The PBMC were isolated from heparinised blood by density gradient separation. Briefly, after sedimentation at rt for 1 h, leukocyte-rich plasma was layered onto LSM 1077 (density gradient solution, PAA, Pasching, Austria) and centrifuged (1000 × g, rt, for 30 min). Interphase PBMC were harvested and washed three times in PBS (500/250/150 × g, rt, 10 min). Cells were counted using the Cellometer Auto T4 cell-counting system (Nexcelom Bioscience, Massachusetts, USA).

### Cell culture

A total of 2 × 10^6^ PBMC per well were seeded in sterile cell culture 12-well plates and incubated for 12 h in a humidified atmosphere at 37 °C (5 % CO_2_ in air) in 1 ml culture medium [(Roswell Park Memorial Institute: RPMI, Biochrom AG, Berlin, Germany) supplemented with 10 % heat-inactivated foetal calf serum (FCS; PAA Laboratories GmbH, Pasching, Austria) and penicillin 100 U/ml, streptomycin 0.1 mg/ml (PAA)] (medium only), supplemented with 1 μg/ml lipopolysaccharide (LPS; #L2755, Sigma-Aldrich, Munich, Germany) (LPS-stimulated), or in medium supplemented with phorbol 12-myristate 13-acetate (PMA; 50 ng/ml) and ionomycin (1.34 μM) (Cell stimulation cocktail 500×, eBioscience, Frankfurt, Germany) (PMA/ionomycin-stimulated). Rather high concentrations of PMA/ionomycin were chosen after preliminary experiments (data not shown) to obtain positive controls of cytokine secretion for each individual.

After incubation, cell-free supernatants were obtained and stored at −80 °C until further analysis. Thawing was performed at 37 °C immediately prior to the cytokine determinations.

Cells in medium only were used to evaluate spontaneous cytokine release. Lipopolysaccharide stimulation was used to evaluate TLR4-response [35] and PMA/ionomycin stimulation to assess cytokine induction by non-specific stimulation [36–38] as positive controls.

### Determination of equine cytokines in cell culture supernatants

Tumour necrosis factor alpha was measured in duplicates by a sandwich ELISA for equine TNFα (Duo Set DY 1814, RnD, Wiesbaden, Germany), performed according to the manufacturer’s protocol with the exception that coating was carried out at 4 °C overnight, as described previously [39]. Absorption was measured using a Synergy 2 instrument (BioTek, Bad Friedrichshall, Germany) and data was analysed by Gene 5 1.11 software (BioTek). The lower and upper limits of detection of the assay were 31.2–2000 pg/ml. Dilutions of standards and supernatant samples (if necessary due to exceeding upper detection limit) were made with PBS containing 1 % bovine serum albumin (BSA; # P3688, Sigma-Aldrich).

Two samples (one value from group A at t6; one value from group C at t24) were excluded from further analysis as the differences measured between TNFα duplicates were excessive.

Interferon alpha, IFNγ, IL-4, IL-10 and IL-17 were measured using a bead-based multiplex assay based on equine-specific monoclonal antibodies on a Luminex 100 System (Luminex, Austin, TX, USA), as validated previously [40]. Limits of detection were as follows: IFNα (12–30,000 pg/ml), IFNγ (10–5000 U/ml), IL-4 (40–80,000 pg/ml), IL-10 (15–35,000 pg/ml) and IL-17 (10–10,000 U/ml).

### Examination of messenger ribonucleic acid

#### Preparation of messenger ribonucleic acid from blood samples

Ribonucleic acid was isolated from whole blood in PAXgene blood RNA tubes with the PAXgene blood RNA Kit (Qiagen GmbH, Hilden, Germany). Isolation was performed according to the manufacturer’s instruction. The RNA concentration of the resulting solution was measured by automated electrophoresis (Experion System, Bio-Rad Laboratories GmbH, Munich, Germany) using the Experion RNA StdSens Starter Kit (Bio-Rad Laboratories GmbH), according to the manufacturer’s instructions.

### Preparation of messenger ribonucleic acid of skin samples

Punch biopsies of 4 mm diameter were transferred into 300 μl RLT buffer (Qiagen GmbH) and homogenised with TissueLyser II (30 Hz, four times for 5 min each, Qiagen GmbH). Subsequently, the RNA was extracted using the RNeasy Fibrous Tissue Mini Kit (Qiagen GmbH), according to the manufacturer’s instructions. The resulting RNA solution was used for analysis by quantitative PCR (qPCR).

### Transcription into cDNA

In order to perform the qPCR, 100 ng RNA diluted to a final volume of 10 μl with DNase/RNase-free water was transcribed into cDNA. SuperScriptII RT (Invitrogen, Karlsruhe, Germany) was used in combination with RNaseOUT Recombinant Ribonuclease Inhibitor (Invitrogen), according to the manufacturer’s instructions.

The resulting RNA solutions were stored at −80 °C and the cDNA solutions at −20 °C.

### Standard series for qPCR

Target-specific primers were designed for conventional and qPCR using the National Centre for Biotechnology Information primer blast in observance of the Equus caballus genome, and were produced by Eurofins MWG Operon, Ebersberg (Table 2). In order to perform the real-time PCR based on SYBR green, standard dilutions were produced for absolute quantification of the cDNA copy numbers.

**Table 2:** qPCR settings

Cytokine	Accession number	Amplicon size (bp)	Forward primer (5'–>3')	Forward primer volume (μl)	Reverse primer (5'–>3')	Reverse primer volume (μl)	Concentration of primers (pmol/μl)
IL12p35	NM_001082511.1	76	GCTGACAGCCATTGACAAGCT	1.5	TTCAAGGGAGGGCTTTTGTG	4.5	0.5
IL12p40	NM_001082516.1	76	TGCTGTTCACAAGCTCAAGTATGA	1.5	GGGTGGGTCTGGTTTGATGA	1.5	0.5
IL18	NM_001082512.1	124	TGCTGGACCAGTAGAAGACA	1.5	AGGTTCAAGCCTGCCAAAGT	1.5	5
IFNγ	NM_001081949.1	417	GCTGTGTGCGATTTTGGGTT	1.5	CTCAGGTTAGCTTTGGGCGA	4.5	5
CXCL10	NM_001114940.1	153	GACTCTGAGTGGAACTCAAGGAAT	1.5	GTGGCAATGATCTCAACACG	4.5	5

Ribonucleic acid was eluted from equine liver, uterus and PBMC by standard procedures using the RNeasy Plus Mini Kit (Qiagen GmbH). The RNA was reverse transcribed into cDNA, as described previously.

A conventional PCR was performed with target-specific primer and Taq DNA Polymerase (5 U/ μl; Invitrogen). The reaction mixture had a final volume of 20 μl, consisting of 2 μl of 10× reaction mix, 0.4 μl of ROTI-MIX PCR 3 (pH 7; Carl Roth GmbH, Karlsruhe, Germany), 0.6 μl of magnesium chloride (50 mM; Invitrogen), 0.2 μl Taq DNA Polymerase (5 U/μl; Invitrogen), 1.5 μl of forward and 1.5 μl of reverse primer (5 pmol/μl), 12.8 μl DNase/RNase-free water (Sigma-Aldrich, Steinheim, Germany) and 1 μl of the cDNA. All samples were incubated at 95 °C for 10 min followed by 40 cycles of 30 s at 95 °C, 30 s at 56 °C and 45 s at 72 °C. Finally, the mixture was incubated at 72 °C for 10 min and stored at −20 °C.

All samples were analysed on 2.5 % agarose gel with Gel Loading Dye Blue (0.08 μl/ml; New England Biolabs GmbH, Frankfurt am Main, Germany) by electrophoresis. The rather high agarose concentration was chosen due to the best separation, as determined in preliminary experiments (data not shown). When the expected size was reached, the product was isolated from the gel band by the QIAEX II Gel Extraction Kit (Qiagen GmbH), according to the manufacturer’s instructions.

One Shot TOP10 Chemically Competent *E. coli* (Invitrogen) was transformed with the target sequences isolated from the gel bands using the pCR 2.1 TOPO TA Cloning Kit (Invitrogen). The *E. coli* solution was transferred to LB medium (32 g LB agar powder (Lennox; Carl Roth GmbH) in 1 l *Aqua tridest*) breeding plates containing ampicillin (Sigma-Aldrich), 5-Brom-4-chlor-3-indoxyl-β-D-galactopyranosid (Invitrogen) and Isopropyl-β-D-thiogalactopyranosid (Invitrogen). The *E. coli* were cultured for at least 24 h at 37 °C. Afterwards, colonies were transferred into 5 ml liquid LB medium (20 g LB agar (Lennox; Carl Roth GmbH) in 1 l A. tridest.) containing 50 μl ampicillin, and cultured for 16 h at 37 °C and 370 rpm. Subsequently, the plasmids were isolated from transformed *E. coli* by the PureLink Quick Plasmid Miniprep Kit (Invitrogen). The plasmids were sequenced by Sequence Laboratories Göttingen GmbH (Göttingen, Germany) to monitor the success of the transformation. Properly transformed plasmids were linearised using the restriction enzyme ScaI (Thermo-Scientific, Schwerte, Germany).

The concentrations of the resulting solutions were measured by BioPhotometer (Eppendorf, Hamburg, Germany) and the copy number per μl was calculated. Finally, solutions with a known concentration of copy numbers were diluted and used as standard series in the qPCR in duplicate.

### qPCR

Real-time SYBR Green PCR was performed on a StepOnePlus Real-Time PCR System (Applied Biosystems, Darmstadt, Germany) with MicroAmp Fast Optical 96-Well Reaction Plates, 0.1 ml (Applied Biosystems) and MicroAmp Optical Adhesive Film (Applied Biosystems). A real-time PCR reaction mixture was used with 12.5 μl SYBR green (Applied Biosystems), forward and reverse primers (Table 2) and the addition of DNase/RNase-free water to achieve a final volume of 25 μl. A volume of 1 μl of sample cDNA solution was added. In order to quantify the amount of copy numbers, the standard dilution series produced were used for comparison (10^2^–10^6^ copies/μl). All measurements were performed in duplicate. The samples were denatured for 10 min at 95 °C, followed by 40 cycles of 15 s at 95 °C and 60 s at 60 °C. A DNA melting curve analysis was carried out to ensure the production of a single PCR product.

### Skin sampling

Four skin samples were taken 24 h after treatment under local anaesthesia after aseptic preparation of the skin at a distance of 1 cm from each injection site. This was the closest distance possible to achieve four equal biopsy samples and to still be able to close the sites afterwards without a high risk of scar formations. Skin biopsy samples were obtained using 8 mm (for histology) and 4 mm (for qPCR) diameter biopsy punches (Stiefel, Munich, Germany). Biopsy sites were closed aseptically by routine surgical closure.

Punch biopsies for qPCR were snap frozen in 1.5 ml RNAlater (Ambion, Carlsbad, USA) in liquid nitrogen and stored at −80 °C until further analysis.

Biopsies for histological examinations were fixed in 4 % neutral buffered formalin [41] for 24 h and then cut into two halves perpendicular to the surface.

Subsequently, formalin-fixed specimens were rinsed in slowly running tap-water for 6 h. After this, specimens were blinded again and embedded in paraffin for storage at rt until further analysis.

### Histology

Sections of the paraffin-embedded tissues were cut (each 3 μm), mounted on glass slides and dried at 60 °C overnight.

Haematoxylin and eosin (H&E) stainings were prepared of each specimen for a general histopathological examination. One half of each specimen was chosen by the criteria of lowest artefacts, presence of all layers of the skin and best plane of cutting (exact sagittal slices preferred) for further evaluation.

### Evaluation

Histopathological alterations were assessed according to a semi-quantitative score of inflammation (Table 3) separately in each layer of the skin [epidermis (Ep); dermis, papillary layer (*Dpap*) including adnexa; dermis, reticular layer (*Dret*); subcutis]. Inflammatory cell patterns were documented and individual cell types were ranked (0 to 3) according to their proportion of the cell infiltrate.

**Table 3:** Histological scores

Score	Epithelium	Dermis
0	No abnormalities	No leukocytes
1	One small ulcer / a few suspect areas with subepithelial infiltration of leukocytes	A few leukocytes, usually perivascular
2	A few ulcers, but predominantly epithelium without alterations	Some leukocytes, usually perivascular mononuclear cells
3	Some ulcers	Many leukocytes, countable
4	Frequent ulcers, intact epithelium between ulcers	Many leukocytes, uncountable due to overlapping or high-density masking cell edges
5	Many ulcers, no epithelium without alterations	Many leukocytes, uncountable tissue structure masked

Descriptions of histological scores for evaluations of epithelium and dermal layers (papillary and reticular) in skin samples stained with H&E

### Immunohistochemistry

Immunohistochemistry (IHC) was performed on slices of the half of the formalin-fixed paraffin-embedded skin samples chosen. Macrophages and neutrophilic granulocytes were detected by staining of calprotectin, which is expressed by these cells [42]. Equine IL-12 and IL-18 were detected by cross-reactive monoclonal antibodies (mAbs), as described previously [43].

Slices of 3 μm were cut and dried on salined glass slides (Histobond, Marienfeld, Lauda-Königshofen, Germany) at 60 °C overnight. The sections were deparaffinised in xylene and rehydrated in a series of alcohols of descending grades. Endogenous peroxidase was blocked in 0.6 % hydrogen peroxide in 80 % ethanol at rt for 30 min. Sections were rinsed 3 times in PBS at rt for 5 min each.

All the following incubation steps were performed in a moist chamber. Pretreatment for antigen retrieval was performed with MAC 387 for optimum results (Table 4). The sections were incubated with proteinase K (#P2308, 7.0–14.0 U/mg, Sigma Aldrich) diluted in PBS at rt for 30 min. The sections were then rinsed in distilled water twice at rt for 2 min and once in PBS at rt for 5 min. The sections were incubated with heat-inactivated normal goat serum (NGS) diluted 1:5 in PBS for 20 min at rt to block unspecific protein binding.

**Table 4:** Protocols in immunohistochemistry

Target	Antibody	Clone, immuno-globulin subclass	Source	Catalogue no.	Pretreatment for antigen retrieval	Dilution final concentration	Secondary antibody, dilution	Signal amplification	Tissue for positive control	Isotype control
Cal-protectin	Macrophage/MAC 387	MAC 387, mouse IgG1	DCS Immunoline, Hamburg, Germany	MI657C01	Proteinase K 5 μg/ml, in PBS, 30 min	1:400 0.1 μg/ml	IHC Kit MouseLink, HRP Label (DCS Immunoline) enhancer	IHC Kit MouseLink, HRP Label (DCS Immunoline) polymer	Tonsil	Mouse IgG1 (Dako, Hamburg, Germany)
IL-12	Mouse anti-bovine IL-12	CC301, mouse IgG2a	AbD Serotec, Puchheim, Germany	MCA1782	None	1:100 10 μg/ml	Anti-mouse-biotin (vector laboratories, Burlingame, Canada) 1:200	Vectastain Elite ABC Kit (vector laboratories)	Colon	Mouse IgG2a (AbD Serotec)
IL-18	Mouse anti-pig IL-18	5-C-5, mouse IgG1	AbD Serotec	MCA2094	None	1:1000 1 μg/ml	Anti-mouse-biotin (vector laboratories) 1:200	Vectastain Elite ABC Kit (vector laboratories)	Colon	Mouse IgG1 (Dako)

The NGS was decanted and the sections were covered with primary antibodies (Table 4) diluted in PBS with 1 % BSA and incubated at 4 °C overnight. Negative controls were incubated with PBS/BSA only. On the following day, sections were rinsed 3 times in PBS for 5 min at rt (negative controls were handled separately).

The sections were then incubated with biotinylated secondary antibodies diluted in PBS at rt for 45 min, followed by rinsing in PBS and signal amplification with avidin-biotin complex (vector laboratories, Burlingame, Canada), according to the manufacturer’s instructions for interleukins, or a ready-to-use kit (IHC Kit, DCS Immunoline, Hamburg, Germany) was employed, according to the manufacturer’s instructions, for calprotectin as a secondary antibody and signal amplification (Table 4).

After rinsing the sections 3 times in PBS for 5 min, visualisation was performed with the chromogen, 3-amino-9-ethylcarbazole (AEC; Peroxidase-Substrat-Kit AEC, Biologo, Kronshagen, Germany), which was applied according to the manufacturer’s instructions. After incubation for 10 min at rt, the slides were rinsed in PBS for 5 min and in slowly running tap-water for 10 min.

Sections were counterstained in Delafield’s haematoxylin for 2 s and rinsed in running tap-water for 10 min to facilitate identification of specific tissue components.

Slides were mounted with Kaiser’s glycerol gelatine (Merck, Darmstadt, Germany) and cover-slips and left to dry at rt.

Sections were viewed with a Zeiss Axioskop (Carl Zeiss Jena GmbH, Jena, Germany) and images were digitally captured using an Olympus DP Soft Camera (Olympus Deutschland GmbH, Hamburg, Germany).

Distinct red staining of cells was interpreted as a positive reaction of the primary antibodies given that negative controls showed no such staining. Negative controls were run in each batch, and isotype controls and positive controls in other tissues had been performed when establishing the IHC settings used.

### Evaluation of immunohistochemistry-stained specimens

Cells stained positive for the respective targets in IHC were separately evaluated in the different layers of the skin (*Ep*, *Dpap*, *Dret*). Since only a few specimens contained sufficient amounts of subcutis, this layer was not assessed further.

Five fields of view (FOV, 0.1435 mm^2^) with a 20,000 × magnification were chosen randomly in *Dpap* and *Dret*, and positively stained cells were counted in images of these by means of the manual tag function of the Image Pro programme (Media Cybernetics, Inc., Rockville, MD, USA). Epithelia were evaluated descriptively.

### Statistical analysis

Statistical analysis was performed with SAS Analytics Pro (SAS Institute Inc., Cary, NC, USA) version 9.3 or higher. P-values < 0.05 were considered significant.

Cytokine data were log transformed due to log-normal distribution of the values for statistical comparisons. Stimulation ratios (times release) were calculated for each cytokine as cytokine _LPS_/cytokine _medium_ and cytokine _PMA/ionomycin_/cytokine _medium_.

### Determination of baselines as internal controls for systemic parameters

Analysis of variance (ANOVA) was employed with time-of-day as a fixed factor and horses as random factors considering the interaction of time-of-day and horses for the analysis of the influence of sampling times on all systemic parameters measured and calculated before treatment.

For parameters influenced by sampling time, baselines were calculated separately for each sampling time (09:00, 15:00, 21:00, 03:00) as means of all values measured at the respective time before treatment. For parameters independent of sampling time (cytokines in PBMC supernatants), baselines were calculated as means over all measurements before treatment.

All measurements were evaluated in relation to individual baselines. This decision was confirmed by an analysis of the influence of baseline values on measurements after treatment in an ANOVA with respect to interactions of treatment and baseline, which showed significant influences of the baseline measurements.

### Influence of treatment on systemic parameters

The influence of the treatments on systemic parameters in relation to calculated baseline measurements was analysed by univariate (uv) and multivariate (mv) ANOVA over all measurements post-treatment. If influences were significant in both models, they were considered relevant.

Questionable parameters of treatment effect, judged by graphic evaluation and ANOVA results, were analysed in detail for measurements at t12 and t24, SAA as a long-term indicator, for all sampling times. The influences of single treatments were analysed by paired t-tests of time-of-day matched baseline values compared to values at t12 and t24. Comparison of treatment effects of A–D at t12 and t24 was performed by estimated pairwise group differences with corresponding p-values and 95 % confidence intervals derived by analysis of covariance (ANCOVA) methods with baseline as covariate (*t*-test, baseline adjusted), not adjusted for multiplicity.

### Influence of treatment on local parameters

The Wilcoxon signed-rank test was employed for clinical and histological scores to compare treatment and control sample for each treatment and parameter.

The Kruskal-Wallis test was used to detect overall differences between treatments employing differences between *treat* and *ctrl* site for individual horses and Wilcoxon signed-rank test was employed to analyse differences from 0 or comparisons of single treatments.

Data were log transformed for immunohistochemistry parameters, except for IL-12/IL-18 ratios, and ANOVA was performed with a Tukey post hoc test to determine differences between treatments and controls and between different treatments.

### Determination of responders

Parameters statistically significantly affected by treatments (differences to baseline, t-tests) were used to determine responders herein. Thresholds and response periods discriminating responders from non-responders were defined by graphic evaluation of courses of the measurements in each parameter. Horses were regarded as responders if their values exceeded the thresholds in at least three parameters. Responders and parameters with responses exceeding thresholds were counted for each group.

### Influence of treatment on messenger ribonucleic acid expression

The difference of copy numbers of mRNA in *treat* (in blood t12) and *ctrl* samples (in blood t0) was calculated for the analysis of mRNA expression in blood and skin samples. These differences were analysed by Wilcoxon signed-rank test.

### Influences of the horse factors: age, sex, type and colour

Horses of groups which had shown no difference by treatment on respective parameters were analysed together for these parameters. The influences of age were analysed by uv ANOVA. Sex, type and colour influences were analysed by unpaired t-tests.

## Results and discussion

Changes of systemic parameters measured were induced by DNA containing treatments (B, C, D) and occurred within 24 h after injection for all parameters measured, except for SAA, an acute phase protein, which was used as a long-term indicator demonstrating effects by 72 h after injection.

In summary, horses showed elevated rectal temperatures (RT) and increased WBC, with the increase mainly attributable to elevated granulocyte levels, after treatment with DNA complexed with SAINT-18 (B, C, D). These effects were interpreted as signs of a mild systemic inflammatory reaction [44, 45].

### Clinical findings

Treatment with SAINT-18 and MIDGE-Th1 vectors was well tolerated in all horses. Clinical parameters were within normal ranges after treatments, except for elevated RT exceeding 38 °C in seven horses. This matches the findings described for this treatment in horses bearing melanoma [18]. Overall, short-term safety of the treatment can be assumed.

**Fig. 2:**
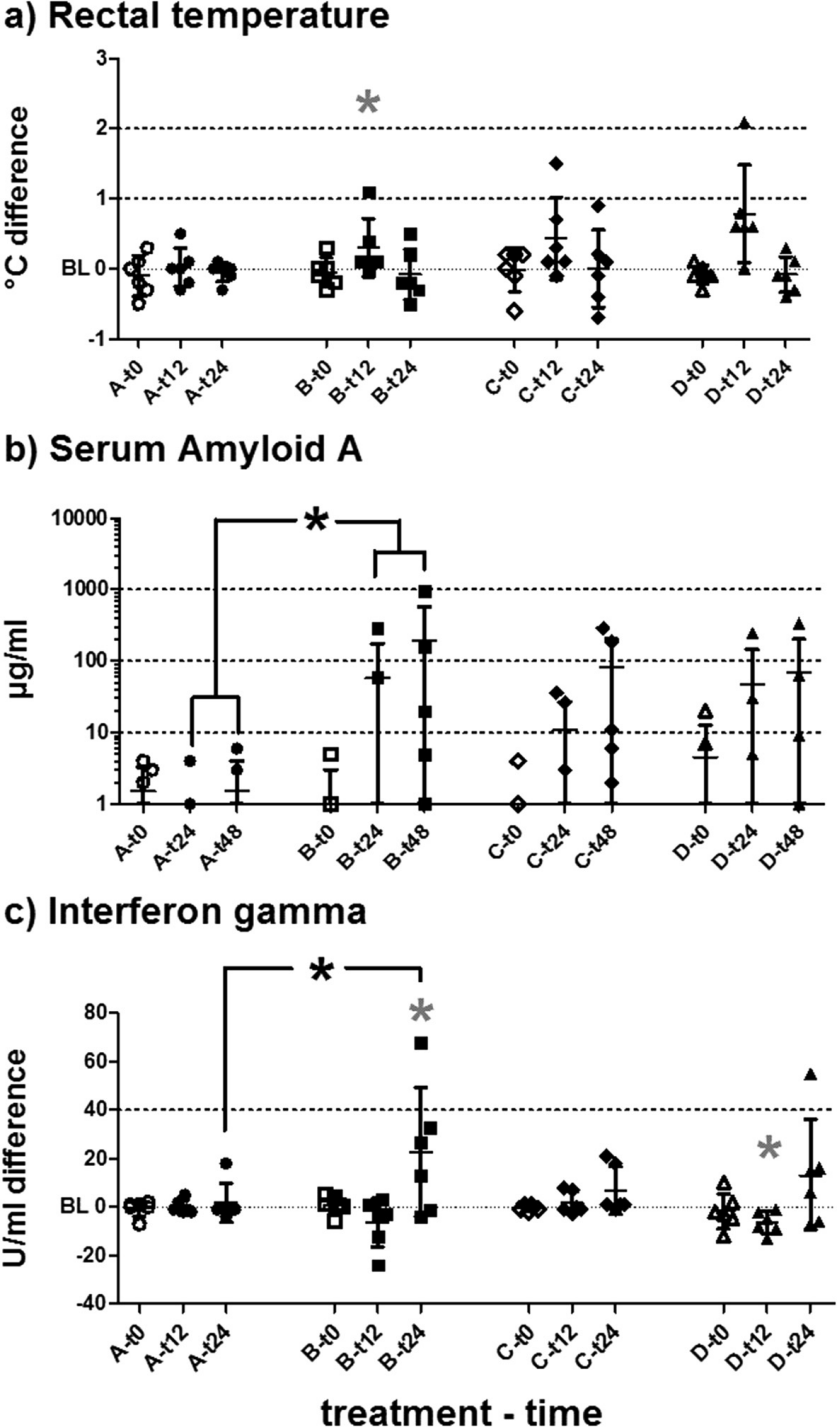
Responses to treatments. **a** Rectal temperature differences to individual time-of-day matched baselines (BL), (**b**) Serum Amyloid A (SAA, absolute concentrations) and (**c**) Interferon gamma (IFNγ) differences to individual baselines in PBMC supernatants (medium only) are plotted in histograms for t0 and two measurements post-treatment. Datasets are marked as (group)-(hours post-treatment) at the X-axis. Horizontal bars represent mean and SD. Grey asterisks (*) represent significant differences from baseline in the respective dataset. Asterisks (*) with brackets (┌ ┌) represent significantly different comparisons. Group B showed values significantly different from baseline in rectal temperatures at t12 and in IFNγ at t24. Horses of this group, furthermore, differed significantly from group A in SAA measurements and IFNγ post-treatment

In comparison to individual time-of-day matched baselines, the RTs of horses in groups B–D were elevated between 12 and 18 h after treatments. This difference reached statistical significance in group B (*t*-test, *p* = 0.0467). However, these elevations of RTs were not significantly different between different treatments (ANCOVA; Fig. 2a).

Rectal temperature increases or development of fever (RT > 38.5 °C) in the healthy horses included were far less frequently observed and tended to last for a shorter time compared to the previous study by Mählmann et al. [18] of horses affected by melanoma. However, since injection of MIDGE-Th1 complexed with SAINT-18 coding for luciferase in five healthy horses had also led to fever in that study, this difference cannot be easily explained by the mere presence or absence of melanoma. Further differences between the studies were the different site of the i.d. injection (peri-tumorally versus neck) and use of local anaesthetics at these sites in the present study. Based on the present data, the authors cannot exclude that the use of subcutaneous lidocaine especially may have inhibited the initial local inflammation at the i.d. injection site and the following increase of RT. Other parameters of potential influence, such as distributions of ages, sexes, breeds and the seasons of treatments, were not statistically significantly different between the previous and the present study. Hence, the reason for this variance between studies remains to be elucidated.

### Blood counts and haemograms

**Fig. 3:**
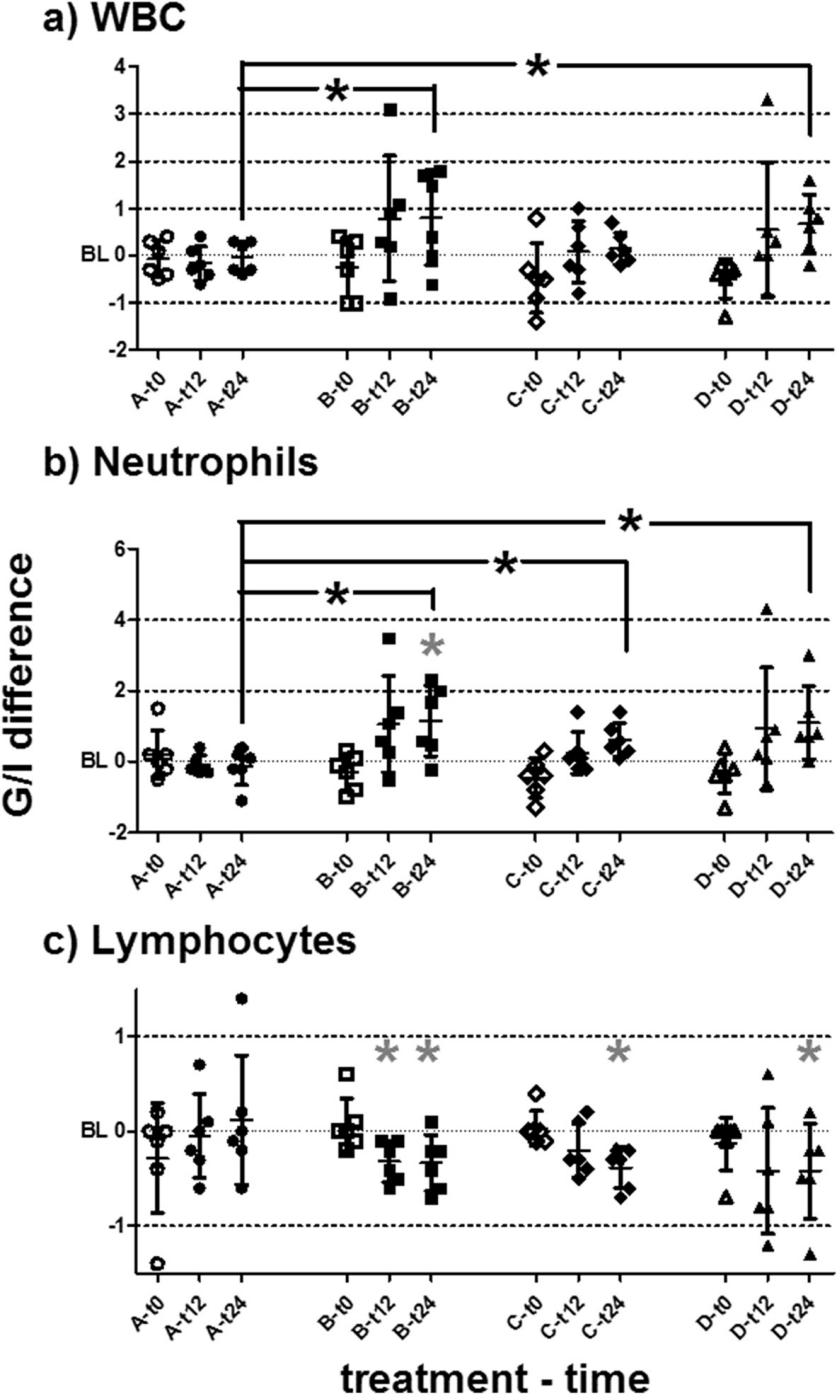
Leucocyte counts. Leucocyte quantities in peripheral blood measured by automated haematology systems are plotted as differences to individual time-of-day matched baselines (BL) for t0, t12 and t24. Datasets are marked as (group)-(hours post-treatment) at the X-axes. Horizontal bars represent mean and SD. Grey asterisks (*) represent significant differences from baseline in the respective dataset. Asterisks (*) with brackets (┌ ┌) represent significantly different comparisons. **a** Increases of WBC induced by B and D at t24 were significantly higher than in A. **b** The increase in neutrophils was significantly different from baseline in B at t24. Group A showed significantly less treatment effects than B, C and D at t24. **c** In comparison to individual baselines, lymphocyte numbers decreased significantly after treatment B at t12 and t24, and at t24 in C and D

All haematologic values measured remained within physiological ranges during all observations, with a tendency towards signs of an acute systemic inflammatory response [45], as follows: WBC increased in comparison to individual time-of-day matched baselines in horses of groups B, C and D between 12 and 36 h after treatment. Treatment effects in group B differed statistically significantly from all other treatments (uv and mv ANOVA, *p* < 0.05). For single comparisons, treatment effects on WBC induced by B and D at t24 were significantly higher than in A (ANCOVA, *p* = 0.0494; 0.0227; Fig. 3a).

Neutrophil numbers increased after all treatments, similar to the WBC effects. The overall effect of treatment on neutrophil numbers was significant (uv and mv ANOVA, *p* < 0.05). However, comparisons of neutrophil numbers to individual baselines showed statistically significant differences 24 h after treatment in group B (*t*-test, *p* = 0.0406) only. Group A showed statistically significantly fewer treatment effects than B, C or D (ANCOVA, *p* = 0.0167, 0.0497 and 0.0184, respectively; Fig. 3b).

Lymphocyte numbers decreased after treatments B, C and D. In comparison to individual baselines, lymphocyte numbers decreased significantly after treatment B at t12 and t24 (*t*-test, *p* = 0.0399 and 0.0111, respectively), and after treatments C and D (*t*-test, *p* = 0.0005 and 0.0276, respectively) only at t24 (Fig. 3c).

The decrease of lymphocyte numbers after the treatments can be explained in the context of an acute systemic inflammatory state of the horses treated with DNA, which may occasionally include lymphopenia, as seen after endotoxin challenge [46]. Decrease in lymphocytes and increase in neutrophils has been demonstrated in mice after systemic administration of complexed plasmid DNA paralleling the present findings in horses [47]. Lymphocyte decrease may be due to extravasation following endothelial activation, as indicated by endothelial swelling and increased perivascular infiltration of lymphocytes in histologic sections of skin samples at locally treated sites in the present study.

### Serum Amyloid A

The horses included usually showed physiological levels of SAA below the lower limit of detection of 5 μg/ml before treatment. Levels of SAA after treatment were highly variable between individual horses of the same treatment group. Despite not reaching statistical significance, SAA in some horses in groups B–D clearly increased 24 to 72 h after treatment. Group B showed statistically significantly higher values after treatment compared to group A (ANOVA uv *p* = 0.0176; mv *p* = 0.0197; Fig. 2b).

All horses had SAA levels below 7 μg/ml at t264, except for one horse (horse # H), which had shown no increase by t72, but had a history of infectious diseases in the herd of its home stable to which it returned at t96.

The increase of SAA indicates an acute phase response, for which it is a sensitive marker in the horse [48]. This again suggests a systemic inflammatory reaction to the DNA treatment in individual horses. Most horses in which SAA increased displayed the rise at t24, but maximums were usually reached at t48 or t72. Serum Amyloid A is known to rise 6 to 12 h after surgery or experimental inflammation and to peak after 48 or even 72 h [34, 49, 50]. This matches the present findings, but may also be due to prolonged response or secondary mechanisms in response to treatments with MIDGE-Th1 vectors, which are detectable long-term after application [51]. Furthermore, biopsy sampling at t24 may have contributed to the acute phase response of the horses. The reaction affecting SAA, however, seems to resolve in a short time, as measurements at t264 no longer displayed increased values.

### Cytokine messenger ribonucleic acid in blood samples

Cytokine expression in peripheral blood samples, determined by qPCR, was not significantly altered between controls (t0) and treatment (t12) in any treatment group or between different treatments. Interferon γ transcripts could not be detected at all in peripheral blood (Additional file 1: Table S1). Insufficient sensitivity of the qPCR assay for *IFNγ* may be assumed, as its absence is contrary to the detection of IFNγ protein. Analysis of mRNA in blood at t12 had been chosen as the horses in the previous study [18] had developed fever 12 to 18 h post-treatment and, thus, systemic effects on mRNA around these time points were expected to be noticed as well. The examination of more time points would be warranted in future studies to track the dynamics of cytokine expressions and to identify later changes.

### Equine cytokines in cell culture supernatants

Cytokines were usually detectable by enzyme-linked immunosorbent assay (ELISA) or bead-based assay in supernatants of *ex vivo*-cultured PBMC (with and without stimuli) and displayed great interindividual variances. Interferon-α was hardly detectable, even after stimulation, and was, thus, excluded from further analysis (Additional file 2: Table S2).

There were no treatment effects detected in individual concentrations and alterations of IL-4 and IL-17 in supernatants of cells cultured without stimuli compared to individual baselines.

Stimulation ratios (e.g. IFNγ _PMA-ionomycin_/IFNγ _medium_) showed high interindividual variances in all cytokines, but no treatment effects were observed.

**Fig. 4:**
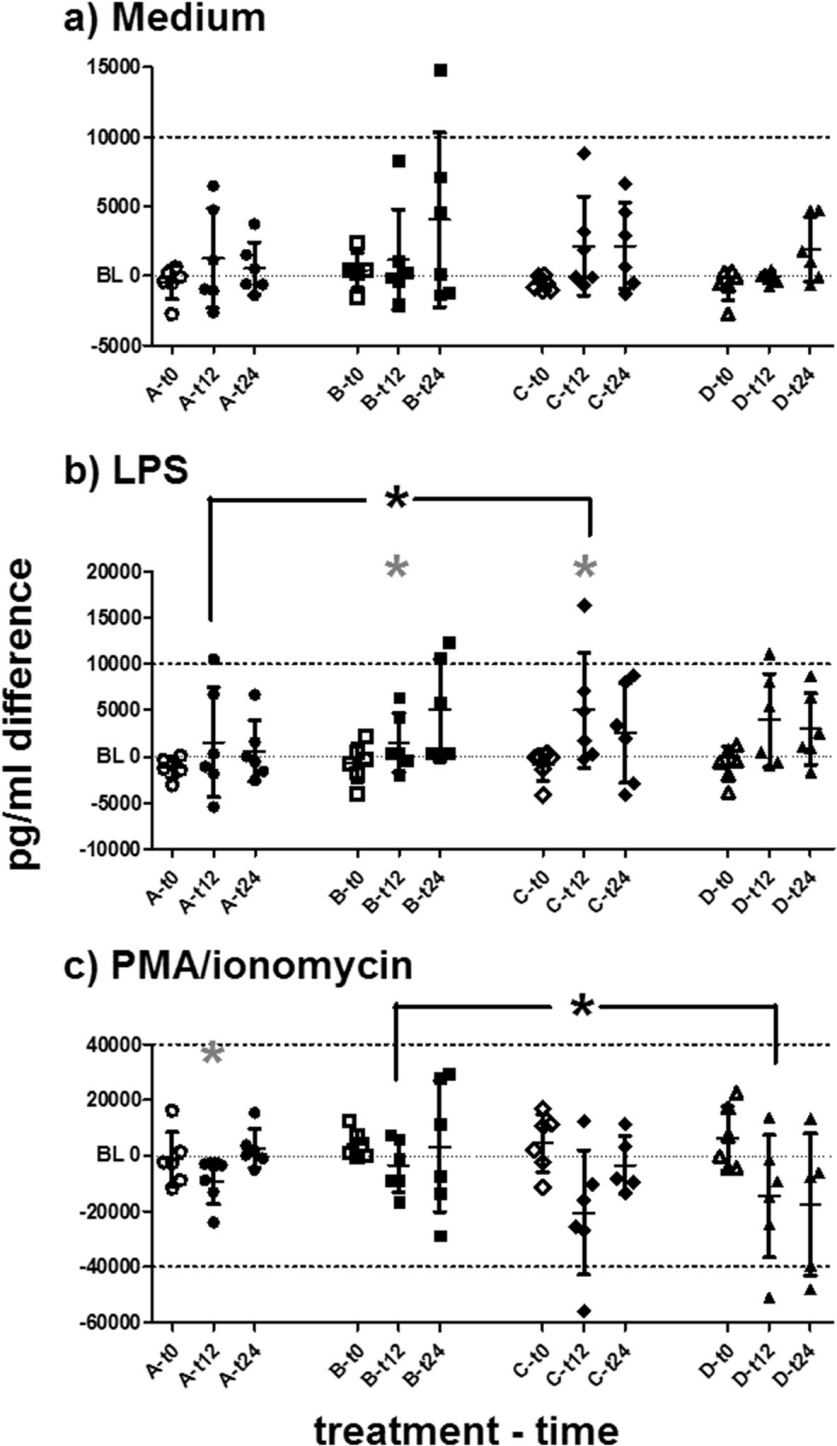
Tumour necrosis factor alpha. Tumour necrosis factor alpha differences to individual baselines in PBMC supernatants measured by ELISA are plotted for releases (**a**) in medium only, (**b**) after LPS stimulation and (**c**) after PMA/ionomycin stimulation for t0, t12 and t24. Datasets are marked as (group)-(hours post-treatment) at the X-axes. Horizontal bars represent mean and SD. Grey asterisks (*) represent significant differences from baseline in the respective dataset. Asterisks (*) with brackets (┌ ┌) represent significantly different comparisons. The increase of TNFα could be statistically noticed in B (t12, medium and LPS), in C (t12, LPS) and in D (t24, medium and LPS). Treatment effects after LPS stimulation at t12 in C or D were higher than in A. Treatments containing DNA (B – D) induced higher TNFα secretion than transfection reagent alone (A)

Tumour necrosis factor alpha (TNFα, in medium only) increased in single horses of all treatment groups compared to individual baselines. *Ex vivo* LPS stimulation of TNFα secretion was mild (mean stimulation ratio = 3.717 times secretion). Changes post-treatment were usually similar in medium and LPS settings. The increase of TNFα after treatments was statistically significant in groups B, C and D. Treatment effects in groups C and D were higher than in A (details given in Fig. 4).

Within 24 h after treatment, levels of IFNγ increased (without *ex vivo* stimuli) only in group B to a mean value statistically significant from group A. Four out of six horses in group B displayed an increase of IFNγ (Fig. 1c). Lipopolysaccharide stimulation of PBMC *ex vivo* resulted in only mild induction of IFNγ (mean stimulation ratio = 2.041 times secretion). Changes after treatments were similar to those in medium settings, but did not reach statistical significance.

**Fig. 5:**
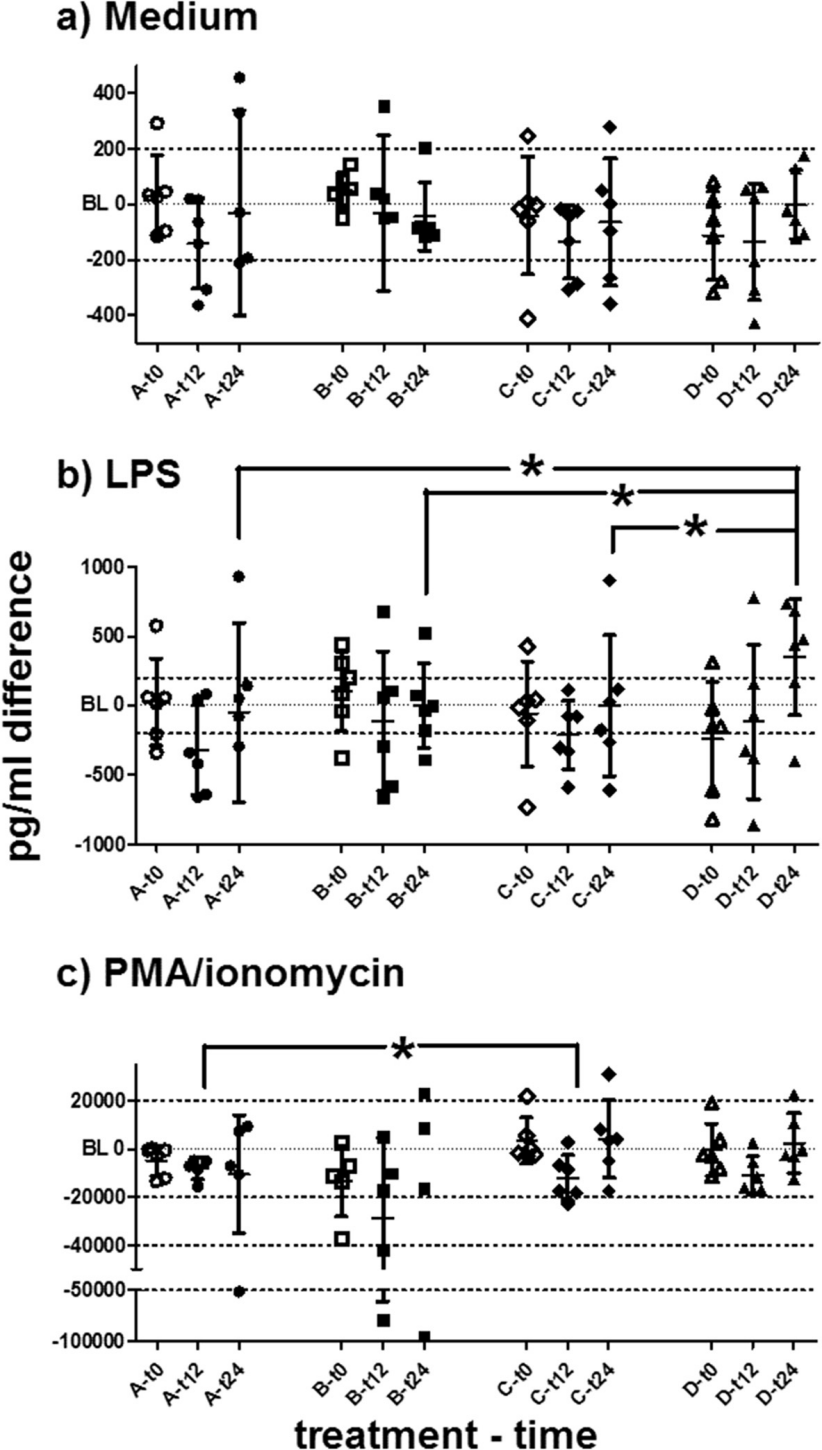
Interleukin 10. Interleukin 10 differences to individual baselines in PBMC supernatants measured by a bead-based assay are plotted for releases (**a**) in medium only, (**b**) after LPS stimulation and (**c**) after PMA/ionomycin stimulation. Data presented at t0, t12 and t24. Datasets are marked as (group)-(hours post-treatment) at the X-axes. Horizontal bars represent mean and SD. Asterisks (*) with brackets (┌ ┌) represent significantly different comparisons. Interleukin 10 decreased after all treatments. Treatment effects after LPS stimulation (b) in group D at t24 (increase) differed significantly from those in A, B and C. After PMA and ionomycin stimulation, treatment effects in C (decrease) differed significantly from those in **a** (near baseline) at t12. The greatest interindividual variances were seen in group **b**

Interleukin-10 in medium settings decreased at t6 after treatment B and at t12 after all other treatments. In general, LPS mildly induced IL-10 secretion *ex vivo* (mean stimulation ratio = 2.821 times secretion). The stimulated IL-10 secretion in PMA and ionomycin settings decreased at t12 in groups B, C and D (Fig. 5).

Detection of cytokine effects proved challenging, as the variance of *ex vivo* cytokine release was high even before treatments. This variance in immune parameters is not unexpected in outbred species such as the horse [52, 53]. However, in comparison to individual baselines, increases of TNFα and IFNγ proteins were systemically induced by treatment with MIDGE-Th1/SAINT-18 (B, C and D), while IL-10 synthesis tended to decrease. These changes after each treatment containing DNA indicate a bias towards a pro-inflammatory immune state [54–58]. It was intended to confirm this at the mRNA level of whole blood leukocytes. However, the qPCR used here was possibly not sensitive enough to detect low numbers of mRNA copies, which may, nevertheless, have been repeatedly translated to detectable amounts of cytokine proteins.

### Systemic effects of DNA treatment

According to the evaluations of systemic parameters in the present study (RT, haemograms, SAA, cytokines in PBMC supernatants), simultaneous i.m. and i.d. treatment with DNA complexed with cationic lipid provokes a systemic immune reaction in horses in accordance with previous reports on inflammation induced by the combination of DNA and cationic transfection reagent in mice [59, 60]. The inflammatory response herein was shown to be independent of transgene expression or CG motif content of the DNA injected, as differences between groups B and D were usually not statistically significant. It could, therefore, be hypothesised that this stimulation of the immune system may be responsible for parts of the antimetastatic effects demonstrated by Mählmann et al. [18] due to subtle modifications of the immune system induced by this antigen-unspecific modulation. However, plasmids containing nonsense DNA alone were not effective in melanoma therapy in previous studies in horses [7, 8], indicating that not just any DNA can be assumed to be effective and complexation with a cationic lipid might be required.

Although most of the recent literature on DNA effects focuses on the immunological effects of CG motifs [26, 61–63], *in vivo* application of complexed DNA herein induced systemic and local effects independent of the CG motif content. It should be mentioned that the CG motif contents of the vectors have been estimated on the basis of counting CG without analysis of the flanking sequences. A detailed analysis was not possible as information on the exact sequences of stimulatory motifs or their allocation to classes are only known for a few oligodeoxynucleotides in the horse [53, 62, 64] and have yet not been completely recognised. In comparison to plasmids, which often carry CG motifs in the backbone, MIDGE vectors only contain CG motifs in their expression cassettes [20, 65]. According to the rather low CG motif content in the MIDGE-Th1 vectors utilised and the absence of statistically significant differences of treatment effects between DNA containing CG and depleted of CG, the authors conclude that signalling via TLR-9 receptors is probably not the primary mechanism of immunostimulatory action of *in vivo* applied complexed MIDGE-Th1 vectors in horses.

Non-nuclear DNA, independent of CG motif and TLR signalling, stimulates innate immune responses, such as pro-inflammatory cytokine production via different pathways, as described more recently [30–33]. The signalling cascades employed are induced by intracellular double-stranded DNA and usually involve cGAS (cyclic GMP-AMP synthase) and STING (stimulator of IFN genes), AIM2 (absent in melanoma 2) and inflammasome activation or ribonucleic acid (RNA) polymerase III and RIG-I (retinoic acid-inducible gene), leading to pro-inflammatory and antiviral immune responses [29, 31, 32]. To the best of the authors’ knowledge, there are no reports of these mechanisms in horses. However, due to their high phylogenetical conservation, they are very likely to exist in horses as well. Thus, although the causative molecular mechanism of the systemic and local immunostimulatory effect independent of the CG motif content of the DNA herein cannot be identified based on the present data, the stimulation of the pathways of the innate immune system by complexed DNA reaching cytosolic compartments described seems likely.

### Responder classification in systemic effects of treatments

The parameters included for responder classification were RT, SAA, neutrophil and lymphocyte counts, TNFα release in response to LPS stimulation and IFNγ release from unstimulated cells (medium only). White blood cell counts, although influenced by treatments, were not included, as this parameter is dependent on neutrophil and lymphocyte counts which were already being considered. Response periods up to t24 were usually chosen, except for SAA (until t72; Table 5). A classification into responders and non-responders was not performed for local parameters evaluated in skin samples because it was not possible to define threshold values by visual inspection due to the lack of graphical dichotomy of the values measured.

**Table 5:** Responders in systemic parameters

Treatment group / horses			A)	# A	# E	# I	# N	# R	# W	B)	# B	# F	# K	# S	# O	# X	C)	# C	# G	# L	# P	# U	# Y	D)	# D	# H	# M	# Q	# V	# Z
Age (years) (groups: mean)			11.1	21	4	11	8	13	9	10.6	5	17	19	7	5	10	10.3	2	8	11	8	20	11	10.9	8	8	10	14	17	8
Sex				fe	fe	mc	mc	mc	mi		fe	mc	mc	fe	mc	mi		mc	mc	mc	mc	mc	mi		fe	fe	mc	mc	mc	mc
Type				ThB	WBl	WBl	WBl	WBl	ThB		ThB	WBl	WBl	WBl	WBl	WBl		ThB	WBl	WBl	WBl	WBl	ThB		ThB	WBl	WBl	WBl	WBl	WBl
Colour				g	g	o	o	o	o		g	o	o	o	o	g		g	o	g	o	o	o		o	g	o	o	g	o
Para-meter	Thre-shold	Period (h)																												
SAA (μg/ml)	10	72	0	−	−	−	−	−	−	3	−	−	+	+	+	−	3	−	−	+	−	+	+	2	+	−	−	−	−	+
RT (°C)^a^	0.5	24	3	+	+	−	−	+	−	3	−	−	+	+	+	−	3	+	−	−	−	+	+	5	+	+	+	+	−	+
Neu (G/l)^a^	1	24	0	−	−	−	−	−	−	3	+	−	−	+	+	−	2	−	−	−	−	+	+	2	+	−	−	−	−	+
Lymph (G/l)^a^	-0.4	24	1	+	−	−	−	−	−	4	−	−	+	+	+	+	2	−	−	−	−	+	+	5	+	+	+	+	−	+
TNFα (pg/ml)^b, LPS^	5000	24	2	+	−	+	−	−	−	3	−	−	+	+	+	−	3	−	−	+	−	+	+	4	+	−	+	+	−	+
IFNγ (U/ml)^b, med^	20	24	0	−	−	−	−	−	−	3	+	−	+	−	−	+	1	−	−	−	−	−	+	2	+	−	−	−	−	+
Responder			1	+	−	−	−	−	−	3	−	−	+	+	+	−	2	−	−	−	−	+	+	4	+	−	+	+	−	+
Responses/group parameters with response			6	3	1	1	0	1	0	19	2	0	5	5	5	2	17	1	0	2	0	5	6	20	6	1	3	3	0	6

Numbers of horses that display values of ≥ threshold given in each cell of group columns; response present (+, threshold reached) or absent (−, threshold not reached) given in each cell of individual horses (IDs)

*fe* female, mare, *mc* male castrated, gelding, *mi* male intact, stallion, *Wbl* Warmblood, *ThB* thoroughbred, *g* grey, *o* other (non-grey), *WBC* white blood cell counts, *Neu* neutrophil counts, *Lymph* lymphocyte counts, *med* medium settings, *LPS* LPS settings, *responder* (response + in min. 3 parameters), *response in groups* at least two horses +

^a^differences to individual time-of-day matched baselines

^b^differences to individual baselines over all times of day

Responders were found in all groups. There were no noteworthy changes of SAA, neutrophil counts or IFNγ in group A. In groups B–D, there were more responses than in A, but no clear differences between types of DNA. Interestingly, not a single grey horse was classified as a responder after treatment with complexed DNA (B, C or D; Table 5).

While some horses displayed systemic effects following DNA application (usually in RT, haematological parameters, acute phase proteins and cytokine secretion), others showed no effects at all. Similar to findings in melanoma patients, responders and non-responders to DNA treatment seem to exist in healthy horses. In accordance with single parameters of systemic evaluations, significant differences between expressing (B) and non-expressing (C) or CG motif-free non-expressing (D) DNA could not be found by the responder classification.

Since no grey horse was classified as a responder, the authors cannot conclude reasonably on the predictive value of the parameters, thresholds and response periods chosen for the responder classification or on anti-tumour effects in grey horses bearing melanomas.

### Evaluation of local treatment effects in clinical and histological examinations

The skin was unaffected and clinical scores were usually classified as grade 0 before treatment. Treatment (A–D injected i.d. locally treated skin samples: *treat*) and control (PBS injected i.d. control skin samples: *ctrl*) sites showed mild oedema (grade 1–2) after treatments. Other alterations were very rare (painfulness: *n* = 2 in *treat* and *ctrl*; redness *n* = 2 in *treat* and *ctrl*; depigmentation = 2 in *ctrl* and *treat*). Clinical scores were not statistically different between control and treatment sites or between different treatments.

The evaluation of skin biopsies stained with H&E revealed ulceration of the epithelia (Additional file 3a–c) and perivascular mixed-cell dermatitis with some additional infiltrating leukocytes in a diffuse pattern, especially in the *Dret*. Inflammatory cells were predominantly of lymphoid origin. Macrophages appeared perivascularly in subepithelial positions proximate to most ulcers and infrequently in diffuse patterns. Neutrophilic granulocytes were randomly seen intravascularly and sometimes found perivascularly and abundantly in a diffuse pattern in higher grade inflammation in *Dret* (Additional files 3a, c and 4a–c).

Scores of inflammation did not differ significantly between treatments and controls or between different treatments in any layer.

Shaving of the skin was performed to optimise the cutting of histological slices of biopsy samples after fixation, as hairs often cause disruption of slices and artefacts in microscopic views. An early time point (t-72) was chosen for shaving to facilitate gross recovery before treatment. Shaving and scrubbing alone led to ulcerative inflammation of the epithelium visible in histologic slides, as shown in six additional healthy horses which underwent shaving and scrubbing without further treatment (Additional file 5). This preparation of the skin may have hidden treatment effects on epithelia, but not on deeper skin layers where the main alterations in response to treatments A–D were expected. However, shaving immediately prior to taking biopsies might have been better. Nevertheless, since the effect of DNA was noted mainly in the dermis, this aspect of the study design may not be of relevance for interpretation of the data.

Local anaesthesia was performed at the i.d. injection sites due to ethical reasons, to facilitate secure intradermal application without complication by defensive actions of the horses and to avoid the need for sedation, as this could have influenced parameters characterizing the systemic immune response. The volume applied is likely to have contributed to the oedema observed. It is, furthermore, possible that the application of local anaesthetics at the i.d. injection sites influenced the immune response to the treatments applied. Local anaesthetics are known to have anti-inflammatory properties [66] and could, thus, have inhibited the inflammatory response. The lidocaine used was applied subcutaneously. Its anaesthetic action is thought to last a maximum of three hours after subcutaneous infiltration [67], allowing the assumption that the majority of the substance should then be cleared from the injection site. MIDGE-Th1 DNA applied intradermally has been described to stay in the dermis long-term, thus, the authors assume that the potential anti-inflammatory effect of lidocaine did not mask the effects of the treatments completely. It still cannot be excluded that local anaesthesia partially influenced the immune response to DNA/SAINT-18.

### Immunohistochemistry results in the skin

#### Calprotectin stained by MAC387

The *strata spinosum, granulosum* and *lucidum*, and hairs’ epithelial sheaths of the stratified squamous epithelium in all specimens were generally immunopositive for calprotectin. Ulcers in the epithelium usually did not stain, except for single infiltrated cells or debris (Additional file 6a, c). Calprotectin positive cells in the dermis were frequent in perivascular locations, but also occurred infrequently in other localisations in a diffuse pattern (Additional files 6b–d and 7a, c). Calprotectin, detected by MAC 387, is a marker for myeloid cells [68] and for epithelial stress [69]. The immunohistochemical staining pattern observed in the present skin samples corresponded well with neutrophilic granulocytes and macrophages identified by H&E staining.

**Fig. 6:**
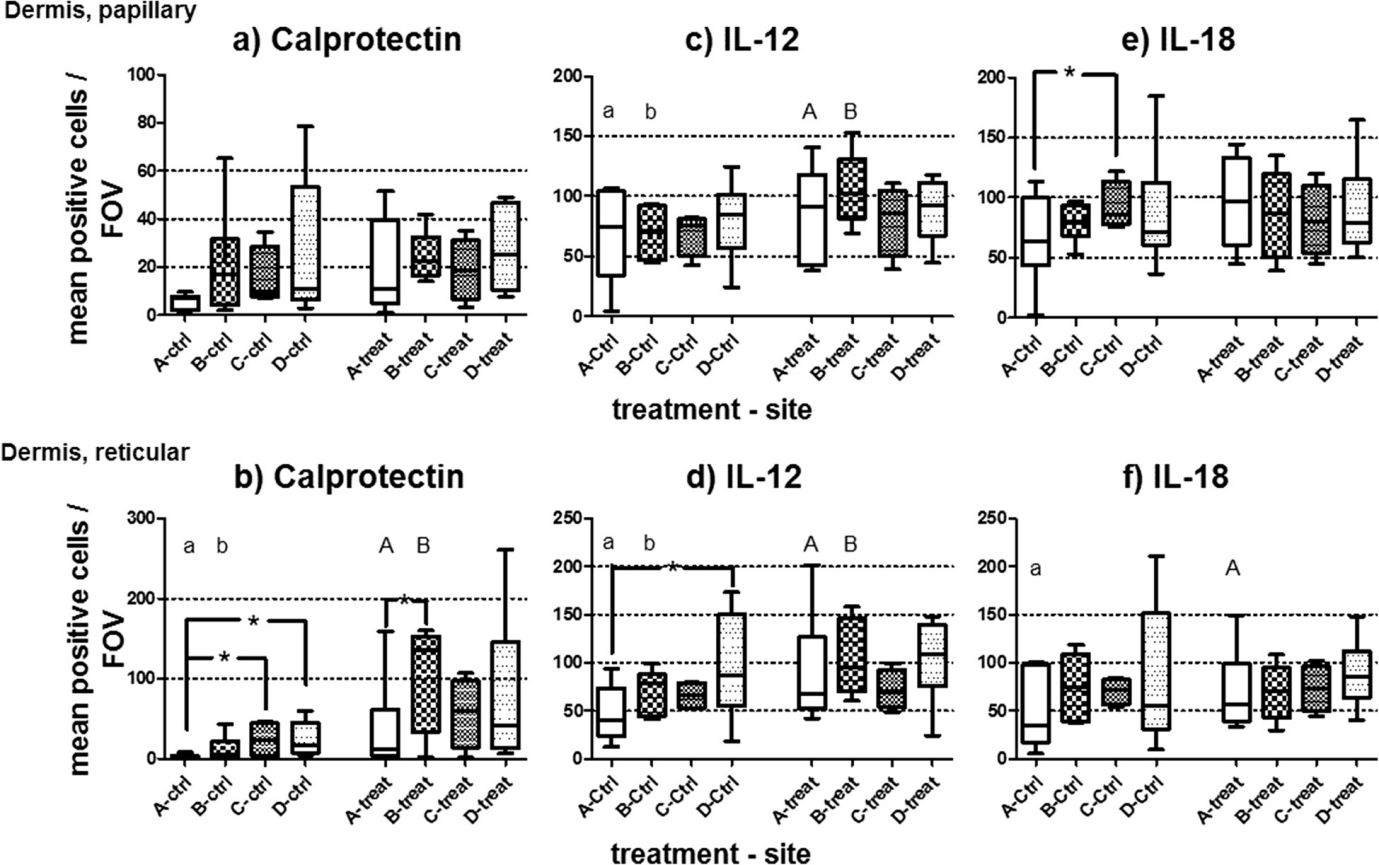
Immunohistochemistry results. Mean positive cells per FOV are plotted for the papillary dermis (*Dpap*, top) and the reticular dermis (*Dret,* bottom) for each treatment (A – D) and site (*Ctrl*: control; *treat*: local treatment) for calprotectin (**a**, **b**), Interleukin (IL)12 (**c**, **d**) and IL-18 (**e**, **f**) determined by immunohistochemistry. Median, quartiles, minimum and maximum plotted for each dataset. Asterisks (*) with brackets (┌ ┌) and same letters (Aa, Bb) represent significantly different comparisons. *Treat* samples overall contained significantly more calprotectin-positive cells and IL-12 positive cells than *ctrl*s in the *Dpap* (a), *and Dret* (b). For single comparisons, this was significant in *Dret* (b) in treatment groups A (Aa) and B (Bb). Control samples of A had significantly fewer calprotectin-, IL-18- and IL-12-positive cells than those of other treatment groups. *Treat* samples of B contained significantly more calprotectin-positive cells in *Dret* than those of A (*)

Overall, *treat* specimens contained more calprotectin-positive cells than *ctrl*s in both layers. For single comparisons, this was significant in the *Dret* in A (Aa) and B (Bb). The *ctrl* samples of B, C and D contained more calprotectin-positive cells than *ctrl* samples of A without reaching significance. In the *Dret*, the *treat* samples of B contained statistically significantly more calprotectin-positive cells than the *treat* samples of A (details given in Fig. 6a, b).

Myeloid cell infiltration reveals that inflammation was induced in both the *treat* and *ctrl* samples. Thus, an acute inflammatory response was induced locally by i.d. injections in general, and by the transfection reagent, SAINT-18, which induced an influx of leukocytes and expression of the cytokines IL-12 and IL-18 (see below). The addition of DNA tended to enhance the inflammatory response, without reaching statistical significance by means of inflammation scores and IHC evaluation. This may be due to the overlapping of the systemic effects causing increased tissue reactivity and effects of the local stimulus by, for example, the pressure of the injection demonstrated in *ctrl* sites.

The treatment schedule closely resembled that of the previous study by Mählmann et al. [18]. The i.m. and i.d. administered portions of the treatments were carried out simultaneously. Therefore, it is not possible to distinguish their contribution to the systemic effects. However, the impact of the route of administration on systemic effects is an interesting aspect that warrants investigation in future studies, considering the routes used and, for example, transdermal application methods used for DNA vaccines [70, 71].

### Local expression of IL-12 and IL-18 proteins in skin samples after treatment

The IL-12 and IL-18 proteins were stained by monoclonal antibodies in IHC for the investigation of transgene expression.

In general, IL-12 and IL-18 were present in the same tissues and cell types in skin samples of the sites treated and of healthy control animals: squamous epithelium, hairs, glands (sebaceous and apocrine), fibrocytes, endothelia (unstained in some bigger vessels), pericytes, muscle cells, nerves (in parts) and leukocytes (Additional files 8, 9, 10, 11, 12 and 13). However, intensity varied, and some cell types (e.g. inflammatory cells) were present more frequently in samples after treatment, resulting in a theoretically higher amount of locally available IL-12 and IL-18.

The *treat* samples contained more IL-12 positive cells than the *ctrl*s. This was statistically significant for single comparisons in treatments A and B in both layers, *Dpap* and *Dret*. Furthermore, the *Dret ctrl*s contained significantly fewer IL-12-positive cells in A than the *Dret ctrl*s in D. The *treat* samples did not differ statistically significantly between different treatments in the absolute numbers of IL-12-positive cells in any layer (details given in Fig. 6c, d ).

There was no statistically significant overall difference in the IL-18-positive cells in the *Dpap* between *treat* and *ctrl*. The *Dpap ctrl*s contained statistically significantly fewer IL-18-positive cells in A than in C. The *treat* samples in *Dret* contained more IL-18-positive cells overall than the *ctrl*s. This was statistically significant in A. The *treat* samples did not differ statistically significantly between different groups in the numbers of IL-18-positive cells (details given in Fig. 6e, f).

### Cytokine and chemokine mRNA in skin biopsies

For further investigation of *in vivo* expression of recombinant *IL-12* and *IL-18*, IL-12 and IL-18 mRNA were measured in skin biopsies by SYBR green qPCR. Furthermore, in order to confirm these and their biologic activity, in addition to the interleukins, the downstream mediator typically induced by IL-12, IFNγ [7] was analysed, as well as a chemokine strongly induced by the latter, CXCL-10 [68] (Additional file 1: Table S1).

The detection of IL-12 expression by qPCR was hampered by the interference of MIDGE- Th1 DNA in the (*treat*) samples, as MIDGE-Th1 vectors present at the site of injection could not be digested completely prior to reverse transcription of mRNA into cDNA (complementary DNA), and PCR primers could not be designed to discriminate between equine recombinant *IL-12* transcripts expressed by MIDGE-Th1 vectors and endogenous *IL-12* transcripts. Consequently, measurement of *IL-12* by qPCR always resulted in positive results and discrimination from false-positive results was not possible.

**Fig. 7:**
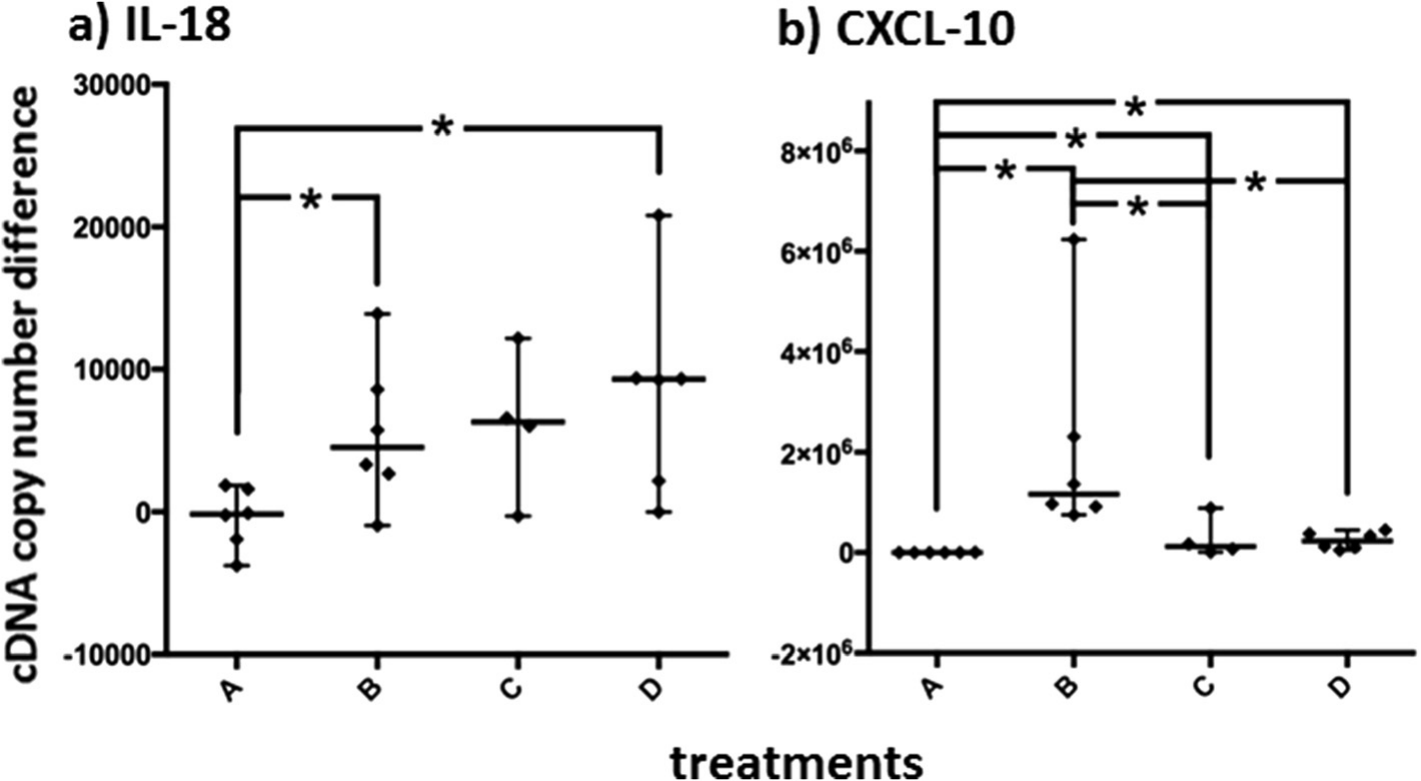
Treatment effects on messenger ribonucleic acid expression in the skin. The *treat*-*ctrl* differences of copy numbers of IL-18 (**a**) and CXCL-10 (**b**) measured by qPCR in skin samples are shown in histograms. Horizontal bars represent medians and ranges. Asterisks (*) with brackets (┌ ┌) represent significantly different comparisons. The expressions of IL-18 increased by local treatment in comparison to *ctrl*s in groups B – D. The expression of CXCL-10 in skin biopsies of the *treat* site was significantly higher overall than in the *ctrl* samples in all treatment groups. The *treat*-*ctrl* difference (b) of copy numbers examined was significantly lower in A than in the other treatment groups. Group B, furthermore, displayed significantly higher *treat*-*ctrl* differences of CXCL-10 copy numbers than C or D

Copy number differences between *treat* and *ctrl* samples of *IL-18* were lowest in group A, which varied statistically significantly from B to D (Fig. 7a).

Copy number differences of *CXCL-10* between *treat* and *ctrl* of individual horses were statistically significantly lower in group A compared to the other groups. Group B, furthermore, showed statistically significantly higher differences of *CXCL-10* mRNA copy numbers between *ctrl* and *treat* than the other groups treated with complexed DNA (C or D) (Fig. 7b).

Similar to the blood samples, *IFNγ* mRNA/cDNA was not detected at all. For IHC, unfortunately, no antibody was available to detect IFNγ protein in comparison. Again, insufficient sensitivity of the qPCR assay for *IFNγ* mRNA/cDNA detection cannot be excluded, as the result on *IFNγ* mRNA levels is contrary to the significant detection of *CXCL-10*.

### Transgene expression

*In vitro* transgene expression after transfection of mammalian cells with MIDGE-Th1 eqIL12 and eqIL18 was proven on the mRNA level [18] and *in vivo* transfection of MIDGE-Th1 vectors was generally confirmed in other species, e.g. in rats, on the protein level as well [72].

Although expression of recombinant IL-12 and IL-18 in group B could not be verified directly by detection of *IL-12* and *IL-18* transcripts, the mRNA of *CXCL-10*, a downstream mediator of IL-12 and IFNγ [73, 74], was significantly elevated in skin samples treated with expressing MIDGE-Th1 (group B, *treat*). As it has been shown that effects on downstream mediators are amplified due to enhancing loops [74], *CXCL-10* mRNA could be detected even if recombinant IL-12 and IL-18, and IFNγ induced by these, are not detectably increased. As CXCL-10 has been shown to be produced after intratumoural injection of IL-12 plasmids in human melanoma [75], subsequent to IFNγ after IL-12 treatment in mice [74] and after transfection of B16 melanoma with IL-18, also in mice [76], it is likely that CXCL-10 is a relevant anti-tumour mediator in melanoma immunotherapy with IL-12/IL-18 DNA also in horses.

Treatment with DNA complexed with SAINT-18 did not lead to vast amounts of cytokines being released by PBMC or expressed by blood leukocytes. The *IL-12* and *IL-18* expression levels were not increased in blood cells after treatments and IL-12 protein could not be detected by a bead-based assay (validated in horses previously [77]) in PBMC supernatants, even after stimulation as evaluated in samples of eight horses of groups A–D, including four responders (data not shown). It can be concluded that circulating leukocytes and tissues influencing these were not transfected to a great extent by expressing DNA vectors, as recombinant *IL-12* and *IL-18* were not statistically significantly elevated in post-treatment blood samples of B and only a moderate increase in the production of IFNγ protein was detected as an induced downstream cytokine of IL-12 and IL-18 [14–16].

However, local treatment with DNA expressing IL-12 and IL-18 (B) induced an increase in IL-12-positive cells in the dermis in comparison to PBS controls. This could be due to local transfection of cells or to induction of endogenous IL-12. This cannot be discriminated herein by means of IHC.

### Influences of horse factors on treatment effects

The authors noted great interindividual variances in treatment effects on most parameters. The following possible influencing factors were evaluated to elucidate the origin of these variances: age, sex (mares vs. geldings; two stallions were excluded from analysis due to limited number), type (breed) and colour of coat.

As treatment differences were usually observed between treatment with (groups B, C and D) and without DNA (group A), horses of groups B, C and D were combined for the analysis of influences on treatment effects.

Mares showed higher increases in WBC (*t*-test, *p* = 0.0068) and neutrophils (*t*-test, *p* = 0.0282), and higher TNFα responses (medium settings; *t*-test, *p* = 0.0172) than geldings (Additional file 14). This is in accordance with the finding of higher plasma levels of TNFα in healthy mares [78] and the authors’ finding of a rather pro-inflammatory tendency of mares compared to geldings when analysing baseline measurements prior to treatments [79].

Thoroughbred type horses showed higher TNFα responses (medium settings; *t*-test, *p* = 0.0240) than WBl type horses (Additional file 14). The breed has previously been shown to influence the cytokine response in TNFα to *in vivo* stimuli [80], but this has not yet been investigated for DNA treatment. The allocation of different breeds to the types of WBl and ThB in the present study may be arbitrary to some degree and does not necessarily represent biological dichotomy. Thus, this limitation should be considered for the judgement of its influence.

Sex and type are known to influence immunological parameters [80–84] and to find such effects herein was not unexpected. As the treatment groups of horses were balanced with respect to these horse parameters, bias of the interpretation of the results concerning treatment effects is not likely.

**Fig. 8:**
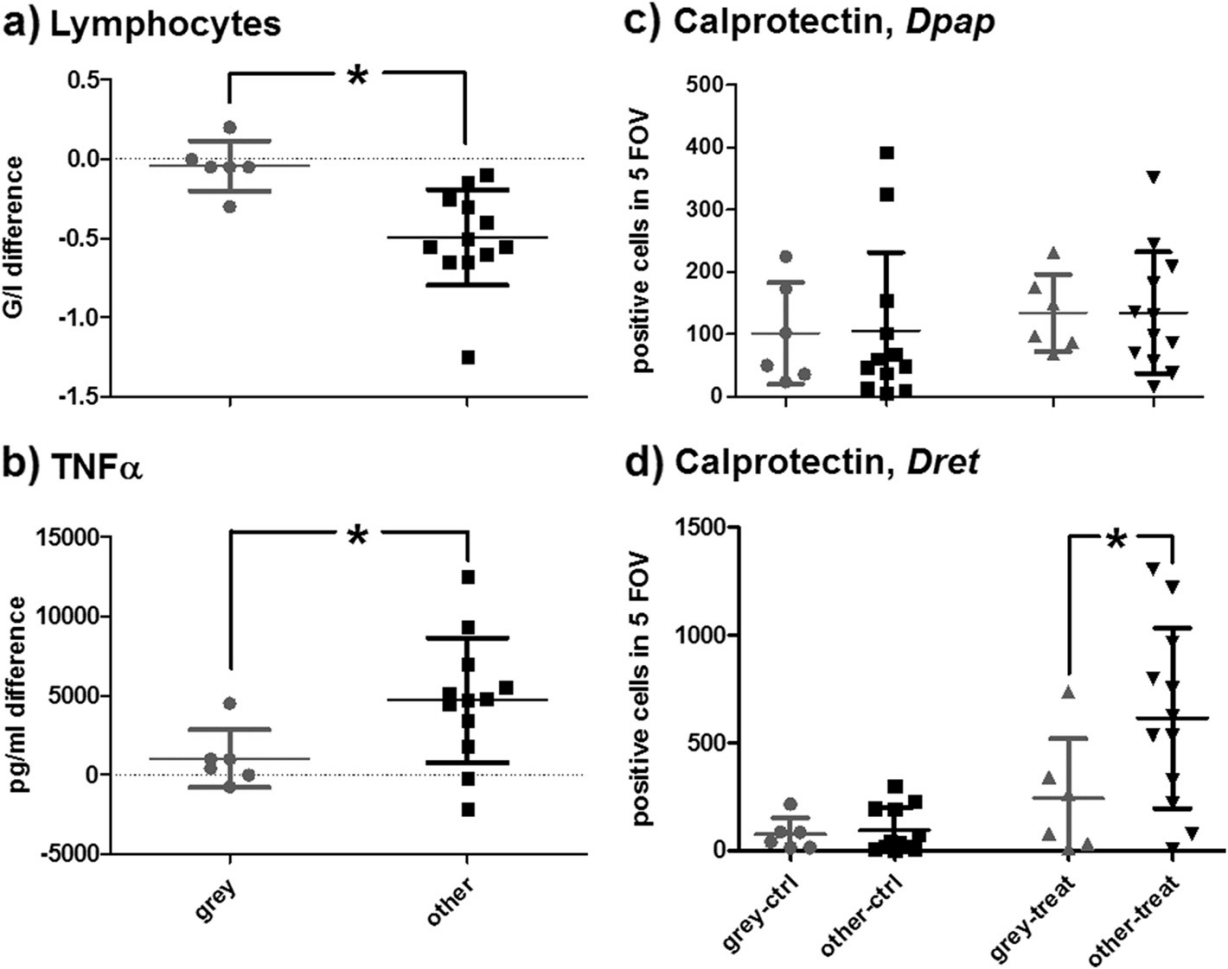
Influence of colour. The results of horses treated with DNA (groups B – D) are summarized. Differences of (**a**) lymphocytes and (**b**) TNFα release after LPS stimulation to individual baselines calculated at t12 and t24 are plotted in histograms for grey horses (grey) and horses of other colours (other). Calprotectin-positive cells in five fields of view (FOV) are plotted (**c**) for the papillary dermis (*Dpap*), and (**d**) the reticular dermis (*Dret*) in histograms for PBS *ctrl* and *treat* sites. Horizontal bars represent mean and SD. Asterisks (*) with brackets (┐ ┐) represent significantly different comparisons. Lymphocyte decreases (a) to individual time-of-day matched baselines were significantly less in grey than in other horses. The TNFα increases to individual baselines in PBMC supernatants were significantly lower in grey than in other horses. Significantly fewer calprotectin-positive cells were found in the *Dret* (b) of *treat* sites of grey horses than in samples of non-grey (other) horses

In addition, there was a relation between the coat colour of the horses and their responses to treatment with DNA. Grey horses showed statistically significantly fewer lymphocyte decreases than horses of other colours (*t*-test, *p* = 0.0037) and fewer increases of *ex vivo* secreted TNFα in response to stimulation with LPS (*t*-test, *p* = 0.0470; Fig. 8). Statistically significantly fewer myeloid (calprotectin-, MAC-387-positive) cells were found (*t*-test, *p* = 0.0413; Fig. 8) in the *Dret* of *treat* samples of grey horses than of non-grey horses, resulting in statistically significantly lower *treat*-*ctrl* differences (*t*-test, *p* = 0.0379).

Furthermore, none of the grey horses included was classified as a responder to DNA treatment with regard to systemic parameters. Therefore, the acute immune reaction of grey horses to DNA treatment seems to be milder than that of non-grey horses as defined by the parameters included in the present report. Some differences between grey and non-grey horses in immunological parameters reported previously, such as different associations of microsatellites and single nucleotide polymorphisms with immune responses to Equine Herpes Virus vaccination [85], match the findings of different immune reactivity reported herein. The milder response of grey horses to DNA complexed with transfection reagent may require stronger stimulation in DNA vaccination, as inflammation enhances DNA uptake and antigen processing [86].

It might even be speculated whether this different acute immunological response contributes to melanoma development in grey horses, as genetic bases of grey coat colour are proposed to have an influence on other organ systems, including cell cycle regulation and immune functions [87–89], which may affect tumour development and immune escape. This is speculative based on the present data of healthy horses, but certainly warrants further investigation.

## Conclusions

Treatment with DNA complexed with the transfection reagent SAINT-18 is immunostimulatory in healthy horses, independent of transgene expression and CG motif content. Grey horses and horses of other colours differ in their systemic and local acute immune reaction to treatment with linear DNA vectors complexed with a cationic lipid. Grey horses seem to be non-responders with respect to non-specific systemic effects of DNA. It cannot be excluded that this characteristic of grey horses may be related to the development of melanoma. Further research elucidating this issue is warranted.

## Additional files

Additional file 1: Table S1. Cytokine cDNA copy numbers (qPCR). Equine cytokine expression measured with absolute quantification by qPCR after transferring mRNA into cDNA. Medians of copy numbers given per μl; t0: before treatment; t12: 12 h after treatment; *ctrl*: local PBS control sample; *treat*: locally treated (A–D) sample; n.d. = not detectable (<100 cDNA copies/μl).2 Additional file 2: Table S2.
*Ex vivo* secreted cytokines. Equine cytokines in PBMC supernatants after 12 h of cell culture: mean concentration (STD); BL: Baseline (mean t-24–t0); t12: 12 h post-treatment; t24: 24 h post-treatment; Detection limits: IFNα (12–30,000 pg/ml), TNFα (31.2–2000 pg/ml), IFNγ (10–5000 U/ml), IL-4 (40–80,000 pg/ml), IL-10 (15–35,000 pg/ml), and IL-17 (10–10,000 U/ml). Additional file 3:
**Histology of epithelia and papillary dermis. Illustration of findings in representative skin samples (H&E staining).** Evaluation was performed for single layers: epithelium (Ep), papillary dermis (*Dpap*), reticular dermis (*Dret*). a) Overview of Ep with two ulcers (arrows) and areas of subepithelial infiltration. Note beginning of ulceration in two additional localisations (dashed arrows). b) Detail of Ep with ulceration (magnification of frame in A). Note cell debris in ulcer (arrow) and subepithelial infiltration of leukocytes (arrowhead) in *Dpap*.c) Inflammation in *Dpap* with adnexa. Distinct infiltration of leukocytes in perivascular localisations (asterisks), while only a few leukocytes are present in a diffuse distribution pattern in other areas. Additional file 4:
**Histology of the reticular dermis. Illustration of findings in representative skin samples (H&E staining) in** ***Dret.*** a) Overview of *Dret* with haemorrhage (arrows). b) Detail of *Dret* with haemorrhage (magnification of A). Note haemorrhage (arrows) and massive perivascular infiltration of leukocytes (asterisks). c) Detail of inflammation in *Dret.* Distinct infiltration of leukocytes in perivascular (asterisks) and intravascular (arrowheads) localisation, while only a few leukocytes are present in a diffuse distribution pattern in other areas. Additional file 5:
**Evaluation of shaving and scrubbing effects on skin irritation.** Description of an additional experiment to evaluate the impact of shaving and scrubbing on skin alterations. Additional file 6:
**Immunohistochemistry for calprotectin. Immunohistochemical localisation of calprotectin (red) in representative skin samples (counterstained with haematoxylin).** a) Overview of Ep and Dermis with low grade inflammation. Calprotectin-positive cells are present in *Dpap* (arrows). In addition, *Ep* and hairs were immunopositive in parts. b) Overview of *Dret* with haemorrhage (arrows). Some leukocytes (asterisks) near and within haemorrhage stained positive for calprotectin (red). c) Epithelial Ulcer (arrow). Subepithelial leukocytes frequently stained calprotectin-positive (red). In addition, parts of the *Ep* (*Str. spinosum and Str. granulosum*) are positive for calprotectin. d) *Dpap* with adnexa. Infiltrating calprotectin-positive (red) leukocytes in perivascular localisation indicate a distinct inflammation. Hairs (H) and excretory ducts of apocrine glands (G) were negative. Additional file 7:
**Detail of calprotectin immunohistochemistry (continued).** Immunohistochemical localisation of calprotectin (red) in representative skin samples (counterstained with haematoxylin), a) *Dret* with perivascular inflammation around larger vessels. Detail of large artery (A) and vein (V), which are surrounded by multiple calprotectin-positive (red) leukocytes. b) *Dret* – Isotype control. Similar localisation as a) with large artery (A) and smaller veins (V), where a perivascular infiltration (asterisks) is visible, but red staining is absent (negative). c) Perineural leukocyte infiltration in *Dret.* Detail of nerve (N) and vessels; note the distinct perivascular and perineural infiltration with calprotectin-positive leukocytes. Additional file 8:
**Immunohistochemistry for Interleukin 12.** Immunostaining for IL-12 (red) illustrated in representative skin samples (counterstaining with haematoxylin). a) Overview of Ep and dermis in inflamed skin. *Ep*, hairs (H) and sebaceous glands (G) stained immunopositive for IL-12 (red). b) Detail of Ep and *Dpap.* The *strata granulosum, spinosum* and *basale* of the stratified squamous epithelium (Ep) showed a cytoplasmic red staining for IL-12. Sebaceous glands (G) and hairs (H) usually stained positive for IL-12. *Musculi arrectores pilorum* (Mm) were also positive for IL-12. In addition, infiltrating leukocytes were frequently positive for IL-12 (arrows). c) Epithelial ulcers. Ulcers (arrows) were unstained in otherwise IL-12-positive stained squamous epithelium. Parts of the fibrocytes (asterisks) in *Dpap* also stained positive for IL-12. d) Isotype control. No immunopositive staining is visible in a similar localisation as in c). Additional file 9:
**Immunohistochemistry for Interleukin 12 in papillary dermis.** Red immunoreactions for IL-12 are shown in representative skin samples with haematoxylin counterstaining. a) Detail of *Dpap* with adnexa. Epithelia of various kinds were usually positive for IL-12, e.g. cortex and epithelial root sheath of hairs (H), and glands and their excretory ducts (asterisks). Please note the patchy staining pattern of the excretory ducts. Endothelia stained positive (arrows) or negative (dashed arrows) for IL-12. Additionally, *Mm. arrectores pilorum* (Mm) stained positive for IL-12. Fibrocytes usually appeared negative for IL-12, but some perinuclear immunopositive staining of fusiform cells in the connective tissue could be noted (circles). b) Detail of *Dpap* illustrating appearance of apocrine glands. Apocrine glands were positive for IL-12 (asterisks), but showed a patchy staining. Epithelial components of hairs (H) were immunopositive, while fibrocytes in this detail were negative for IL-12.c) *Dpap* – detail of subepithelial inflammation. Infiltrating leukocytes can be found perivascularly and are dispersed diffusely in the connective tissue. They frequently show a red perinuclear and cytoplasmic immunostaining for IL-12 (arrows). *Mm. arrectores pilorum* (Mm) and endothelia (dashed arrows) stained positive for IL-12, while fibrocytes were usually negative for IL-12. Epithelium (Ep) and a gland (G) are indicated at the edges of the image (top right and bottom left, respectively). Additional file 10: Immunohistochemistry for Interleukin 12 in reticular dermis. Immunostaining for IL-12 (red) in representative skin samples (counterstaining with haematoxylin). a) Large artery and nerve in *Dret*. Walls and endothelia (dashed arrows) of a large artery (A) and small blood vessels stained positive for IL-12, as did the nerve (N). In addition, infiltrated leukocytes (arrows) were positive for IL-12. Fibrocytes and extracellular matrix were usually negative. b) *Dret* – detail with perivascular inflammation. Leukocytic perivascular infiltration of high degree proximate to artery (A) and vein (V). In addition, vessel walls and endothelia (arrows) infiltrated cells (asterisks) are positive for IL-12. Fibrocytes and extracellular matrix are negative apart from a weak background staining. c) Isotype control *Dret* with artery (A). All tissue components are negative in detail of *Dret*. Additional file 11:
**Immunohistochemistry for Interleukin 18.** Illustration of red IL-18 immunoreaction in representative skin samples (counterstained with haematoxylin). a) Overview of Ep and dermis in mildly inflamed skin. *Ep* (except for ulcers, arrows), hairs (H) and glands (G) stained positive for IL-18. b) Isotype control – overview of Ep and dermis. No immunopositive staining is visible in a similar localisation as in a) in the papillary layer. c) Detail of Ep with ulcer (arrow) and *Dpap* with medium grade leukocyte infiltration. All epithelial tissues [*stratum granulosum, spinosum* and *basale* of the stratified squamous epithelium (Ep), cortex and epithelial root sheath of hairs (arrowheads), sebaceous glands (G)] showed a cytoplasmic immunoreaction for IL-18, while ulcers (arrow) were negative for IL-18. Infiltrating leukocytes (arrowheads) were frequently positive for IL-18. The IL-18 staining of fibrocytes and endothelia (dashed arrows) varied from positive to negative. d) Detail of Ep and *Dpap*, isotype control. A corresponding section to c) did not show any reaction for IL-18. Symbols: stratified squamous epithelium (Ep) with an ulcer (arrow), hairs (cross-section, H), sebaceous glands (G), endothelia (dashed arrows) and leukocyte infiltration (arrowheads). e) Detail of *Dpap* with vessels and excretory ducts of apocrine glands (G). Blood vessel walls and endothelia (dashed arrows) of a larger artery (A) and smaller vessels stained positive for IL-18 (dashed arrows) usually appeared with immunopositive *Lamina media* and endothelia (dashed arrows). Excretory ducts of apocrine glands (G) were positive for IL-18. Fibrocytes and extracellular matrix were usually negative. f) Detail of adnexa in *Dpap.* The epithelial root sheath of a hair (H) and *Mm. arrectores pilorum* (Mm) always stained immunopositive for IL-18, while apocrine glands (G) stained positive, but with some patchy appearance. Fibrocytes and extracellular matrix were usually negative. Additional file 12:
**Immunohistochemistry for Interleukin 18 (continued).** Immunostaining for IL-18 (red) in representative skin samples (counterstaining with haematoxylin). a) *Dret* – detail with perivascular inflammation. Leukocytic perivascular infiltration is visible proximate to artery (A) and vein (V). Vessel walls and endothelia (dashed arrows), as well as infiltrated cells (arrows) are positive for IL-18. Fibrocytes and extracellular matrix are negative, apart from a weak background staining. b) Cross-sectioned nerve (N) in subcutis. Most parts of the nerve (N) stained immunopositive. In addition, adjacent capillary endothelia were positive for IL-18 (dashed arrows). c) Detail of infiltrated cells in *Dpap*. Infiltrating subepithelial immune cells were usually positive for IL-18 (arrowheads), as well as capillary endothelia (dashed arrows). Positive stratified squamous epithelium (Ep) and negative basis of an ulcer (arrow) are visible at the top edge of the detail. Additional file 13:
**Staining characteristics of IL-12 and IL-18 in IHC.** Detailed description of IL-12 and IL-18 stainings of skin biopsies. Additional file 14:
**Influence of sex and type on systemic parameters.** Differences to individual baselines of a) WBC, b) neutrophils and c, d) TNFα release (in medium only) of horses treated with DNA (groups B–D) calculated at t12 and t24 are plotted in histograms for mares and geldings (a–c) or Thoroughbreds (ThB) and Warmbloods (WBl) (d). Horizontal bars represent mean and SD. Asterisks (*) with brackets (┌ ┐) represent significantly different comparisons. The WBC, neutrophil and TNFα increases to individual baselines after treatments with SAINT-18 formulated DNA were significantly higher in mares than in geldings. Increases of TNFα releases were significantly higher in ThB than in WBl.

## Abbreviations

#: Horse identification (letters)
AIM2: Absent in melanoma 2
ANCOVA: Analysis of covariance
ANOVA: Analysis of variance
BSA: Bovine serum albumin
cDNA: Complementary DNA
cGAS: Cyclic GMP-AMP synthase
*ctrl*: Control skin samples, locally treated with PBS
CXCL: Chemokine (C-X-C motif) ligand
DNA: Deoxyribonucleic acid
*Dpap*: Papillary dermis
*Dret*: Reticular dermis
*E. coli*: *Escherichia coli*
EDTA: Ethylenediaminetetraacetate
ELISA: Enzyme-linked immunosorbent assay
FOV: Fields of view
H&E: Haematoxylin and eosin
Hct: Haematocrit
i.d.: Intradermal
i.m.: Intramuscular
IFN: Interferon
IHC: Immunohistochemistry
IL: Interleukin
ILRAP: IL-1beta receptor antagonist protein
LB: Luria-Bertani medium
LPS: Lipopolysaccharide
mAb: Monoclonal antibody
MIDGE: Minimalistic immunologic defined gene expression
mRNA: Messenger ribonucleic acid
mv: Multivariate
NGS: Normal goat serum
PBMC: Peripheral blood mononuclear cells
PBS: Phosphate buffered saline
PMA: Phorbol 12-myristate 13-acetate
qPCR: Quantitative PCR
RIG-I: Retinoic acid-inducible gene
RNA: Ribonucleic acid
rpm: Rounds per minute
RPMI: Roswell Park Memorial Institute
RT: Rectal temperature
rt: Room temperature
SAA: Serum amyloid A
SD: Standard deviation
STING: Stimulator of IFN genes
t(hours): Time post-treatment
t-(hours): Time pretreatment
t0: Time immediately prior to treatment
ThB: Thoroughbred
TLR: Toll-like receptor
TNF: Tumour necrosis factor
TPP: Total plasma protein
*treat*: Locally treated skin samples
uv: Univariate
WBC: White blood cell counts
WBl: Warm blood
