# Supplementary material for: Immune response of healthy horses to DNA constructs formulated with a cationic lipid transfection reagent
Source: BMC Vet Res. 2015 Jun 23;11:140. doi: 10.1186/s12917-015-0452-3 (PMC4476236; doi:10.1186/s12917-015-0452-3)
Supplement: Additional file 1: Table S1. — Cytokine cDNA copy numbers (qPCR). Equine cytokine expression measured with absolute quantification by qPCR after transferring mRNA into cDNA. Medians of copy numbers given per μl; t0: before treatment; t12: 12 h after treatment; ctrl: local PBS control sample; treat: locally treated (A–D) sample; n.d. = not detectable (<100 cDNA copies/μl).2 [file 12917_2015_452_MOESM1_ESM.docx]

**Additional file 1: Table S1 cytokine cDNA copy numbers (qPCR)**

| **Tissue** | | **Blood** | | | | | | | | **Skin** | | | | | | | |
| --- | --- | --- | --- | --- | --- | --- | --- | --- | --- | --- | --- | --- | --- | --- | --- | --- | --- |
| **Treatment** | | **A** | | **B** | | **C** | | **D** | | **A** | | **B** | | **C** | | **D** | |
| **Cytokine** | | t0 | t12 | t0 | t12 | t0 | t12 | t0 | t12 | *ctrl* | *treat* | *ctrl* | *treat* | *ctrl* | *treat* | *ctrl* | *treat* |
| **IL-12p35** |  | 1,715 | 1,425 | 852 | 2,028 | 2,656 | 2,007 | 1,452 | 1,645 |  |  |  |  |  |  |  |  |
| **IL-12p40** |  | 45 | 113 | 123 | 132 | 118 | 119 | 97 | 87 |  |  |  |  |  |  |  |  |
| **IL-18** |  | 391 | 526 | 1,349 | 2,889 | 1,899 | 2,345 | 1,484 | 2,070 | 4256 | 4444 | 3,911 | 9,217 | 5,123 | 10,548 | 5,991 | 13,317 |
| **IFNγ** |  | n.d. | n.d. | n.d. | n.d. | n.d. | n.d. | n.d. | n.d. | n.d. | n.d. | n.d. | n.d. | n.d. | n.d. | n.d. | n.d. |
| **CXCL-10** |  | 1,079 | 515 | 726 | 1,884 | 2,108 | 2,674 | 1,297 | 4,281 | 2,167 | 626 | 3,045 | 1,167,155 | 845 | 120,600 | 3,099 | 230,462 |

Equine cytokine expression measured with absolute quantification by qPCR after transferring mRNA into cDNA. Medians of copy numbers given per µl; t0: before treatment; t12: 12 h after treatment; *ctrl*: local PBS control sample; *treat*: locally treated (A – D) sample; n.d. = not detectable (<100 cDNA copies/µl).
