# Supplementary material for: Immune response of healthy horses to DNA constructs formulated with a cationic lipid transfection reagent
Source: BMC Vet Res. 2015 Jun 23;11:140. doi: 10.1186/s12917-015-0452-3 (PMC4476236; doi:10.1186/s12917-015-0452-3)
Supplement: Additional file 2: Table S2. — Ex vivo secreted cytokines. Equine cytokines in PBMC supernatants after 12 h of cell culture: mean concentration (STD); BL: Baseline (mean t-24–t0); t12: 12 h post-treatment; t24: 24 h post-treatment; Detection limits: IFNα (12–30,000 pg/ml), TNFα (31.2–2000 pg/ml), IFNγ (10–5000 U/ml), IL-4 (40–80,000 pg/ml), IL-10 (15–35,000 pg/ml), and IL-17 (10–10,000 U/ml). [file 12917_2015_452_MOESM2_ESM.docx]

**Additional file 2: Table S2 *Ex vivo* secreted cytokines**

|  |  | **All** |  | **A** |  | **A** | **A** | **B** |  | **B** | **B** | **C** |  | **C** | **C** | **D** |  | **D** | **D** |
| --- | --- | --- | --- | --- | --- | --- | --- | --- | --- | --- | --- | --- | --- | --- | --- | --- | --- | --- | --- |
| **Cytokine** | *setting* | **BL mean** | *BL STD* | **BL mean** | *BL STD* | t12 mean | t24 mean | **BL mean** | *BL STD* | t12 mean | t24 mean | **BL mean** | *BL STD* | t12 mean | t24 mean | **BL mean** | *BL STD* | t12 mean | t24 mean |
| **IFNα (pg/ml)** | *medium* | **1** | *3* | **2** | *5* | 1 | 8 | **0** | *1* | 0 | 2 | **0** | *1* | 2 | 0 | **1** | *2* | 0 | 3 |
| **IFNα (pg/ml)** | *PMA/ionomycin* | **16** | *15* | **13** | *10* | 10 | 12 | **16** | *12* | 14 | 18 | **17** | *22* | 11 | 15 | **16** | *13* | 10 | 11 |
| **TNFα (pg/ml)** | *medium* | **2122** | *2560* | **2686** | *2938* | 3785 | 3192 | **2881** | *3457* | 3926 | 7443 | **1471** | *1365* | 3325 | 4367 | **1421** | *1610* | 1364 | 3170 |
| **TNFα (pg/ml)** | *LPS* | **4435** | *3874* | **4168** | *4107* | 5694 | 4761 | **6081** | *4924* | 7581 | 11039 | **3970** | *2762* | 8966 | 7762 | **3460** | *2926* | 7563 | 6623 |
| **TNFα (ng/ml)** | *PMA/ionomycin* | **36.4** | *23.5* | **33.6** | *26.3* | 24.4 | 36.3 | **44.8** | *22.2* | 41.2 | 48.2 | **33.3** | *21.4* | 20.2 | 37.7 | **33.9** | *23.1* | 19.4 | 24.9 |
| **IFN**γ **(U/ml)** | *medium* | **13** | *12* | **9** | *5* | 10 | 12 | **20** | *19* | 13 | 42 | **7** | *2* | 9 | 14 | **17** | *10* | 10 | 30 |
| **IFN**γ **(U/ml)** | *LPS* | **25** | *32* | **13** | *7* | 13 | 28 | **40** | *38* | 31 | 76 | **10** | *6* | 15 | 32 | **36** | *43* | 16 | 70 |
| **IFN**γ**(U/ml)** | *PMA/ionomycin* | **961** | *319* | **926** | *264* | 831 | 906 | **1052** | *178* | 899 | 1049 | **932** | *384* | 566 | 1052 | **932** | *400* | 723 | 946 |
| **IL-4 (pg/ml)** | *medium* | **552** | *813* | **280** | *305* | 308 | 194 | **448** | *551* | 563 | 665 | **1030** | *1335* | 1334 | 586 | **452** | *466* | 281 | 267 |
| **IL-4 (ng/ml)** | *PMA/ionomycin* | **124.5** | *73.2* | **111.7** | *53.0* | 96.2 | 106.8 | **133.1** | *66.7* | 109.5 | 135.9 | **119.4** | *90.0* | 86.1 | 142.8 | **134.0** | *79.9* | 101.6 | 118.9 |
| **IL-10 (pg/ml)** | *medium* | **466** | *297* | **552** | *386* | 411 | 521 | **459** | *303* | 426 | 415 | **435** | *230* | 301 | 370 | **417** | *244* | 282 | 416 |
| **IL-10 (pg/ml)** | *LPS* | **1053** | *679* | **1128** | *935* | 808 | 1078 | **1067** | *640* | 955 | 1069 | **959** | *375* | 748 | 960 | **1059** | *683* | 944 | 1409 |
| **IL-10 (ng/ml)** | *PMA/ionomycin* | **33.8** | *30.2* | **32.0** | *26.7* | 33.8 | 59.5 | **55.2** | *43.3* | 35.4 | 103.3 | **22.8** | *14.4* | 11.0 | 26.8 | **25.2** | *16.8* | 14.3 | 27.6 |
| **IL-17 (U/ml)** | *medium* | **154** | *224* | **248** | *314* | 243 | 196 | **240** | *252* | 180 | 212 | **53** | *80* | 30 | 46 | **74** | *68* | 38 | 71 |
| **IL-17 (1000 U/ml)** | *PMA/ionomycin* | **27.8** | *14.0* | **25.6** | *13.7* | 22.3 | 24.2 | **29.7** | *13.5* | 23.0 | 30.2 | **27.4** | *13.8* | 16.8 | 30.9 | **28.5** | *15.3* | 19.4 | 22.1 |

Equine cytokines in PBMC supernatants after 12 h of cell culture: mean concentration (STD); BL: Baseline (mean t-24 – t0); t12: 12 h post-treatment; t24: 24 h post-treatment; Detection limits: IFNα (12 – 30,000 pg/ml), TNFα (31.2 – 2,000 pg/ml), IFNγ (10 – 5,000 U/ml), IL-4 (40 – 80,000 pg/ml), IL-10 (15 – 35,000 pg/ml), and IL-17 (10 – 10,000 U/ml).
