# Supplementary figures and images for: Immune response of healthy horses to DNA constructs formulated with a cationic lipid transfection reagent
Source: BMC Vet Res. 2015 Jun 23;11:140. doi: 10.1186/s12917-015-0452-3 (PMC4476236; doi:10.1186/s12917-015-0452-3)

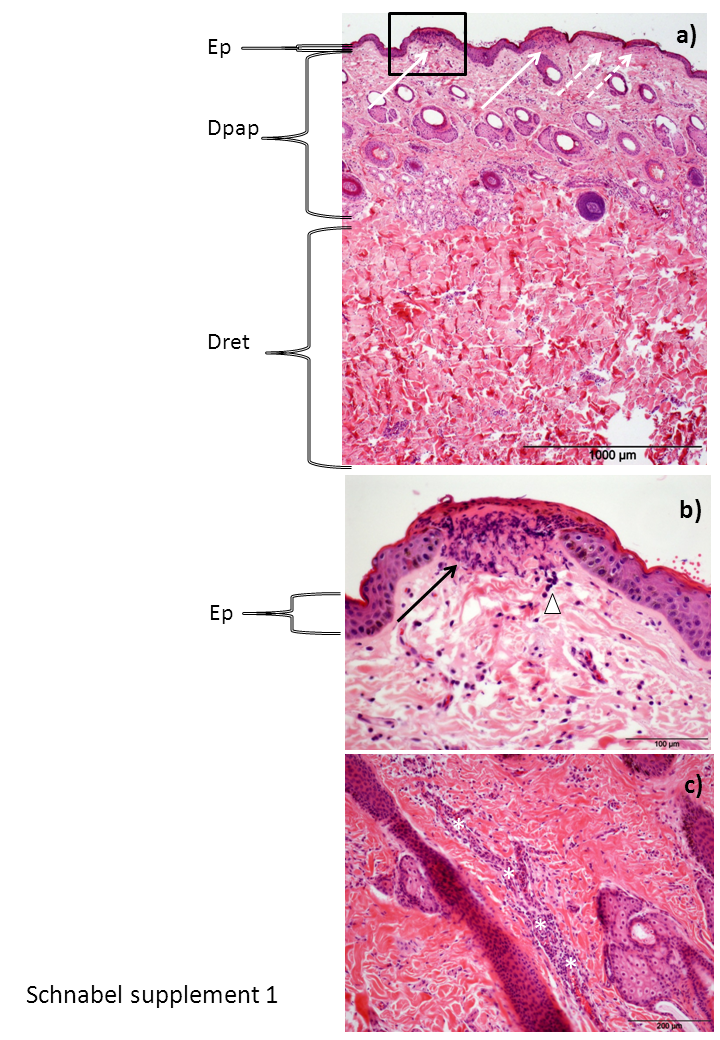

Supplement: Additional file 3: — Histology of epithelia and papillary dermis. Illustration of findings in representative skin samples (H&E staining). Evaluation was performed for single layers: epithelium (Ep), papillary dermis (Dpap), reticular dermis (Dret). a) Overview of Ep with two ulcers (arrows) and areas of subepithelial infiltration. Note beginning of ulceration in two additional localisations (dashed arrows). b) Detail of Ep with ulceration (magnification of frame in A). Note cell debris in ulcer (arrow) and subepithelial infiltration of leukocytes (arrowhead) in Dpap.c) Inflammation in Dpap with adnexa. Distinct infiltration of leukocytes in perivascular localisations (asterisks), while only a few leukocytes are present in a diffuse distribution pattern in other areas. [file 12917_2015_452_MOESM3_ESM.png]

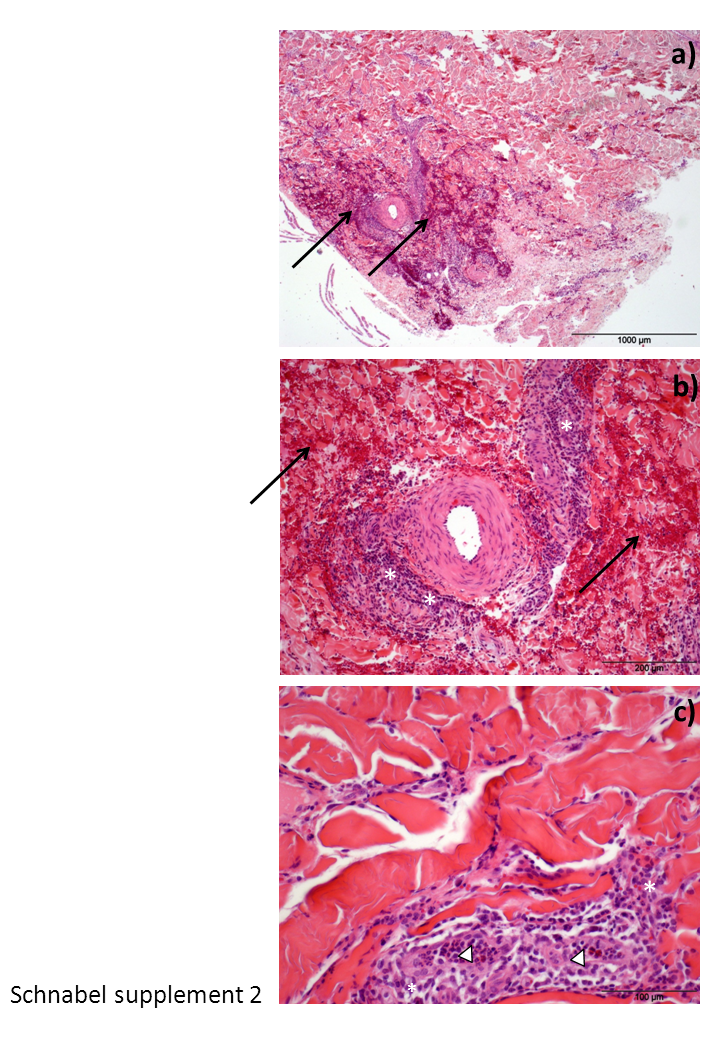

Supplement: Additional file 4: — Histology of the reticular dermis. Illustration of findings in representative skin samples (H&E staining) in Dret. a) Overview of Dret with haemorrhage (arrows). b) Detail of Dret with haemorrhage (magnification of A). Note haemorrhage (arrows) and massive perivascular infiltration of leukocytes (asterisks). c) Detail of inflammation in Dret. Distinct infiltration of leukocytes in perivascular (asterisks) and intravascular (arrowheads) localisation, while only a few leukocytes are present in a diffuse distribution pattern in other areas. [file 12917_2015_452_MOESM4_ESM.png]

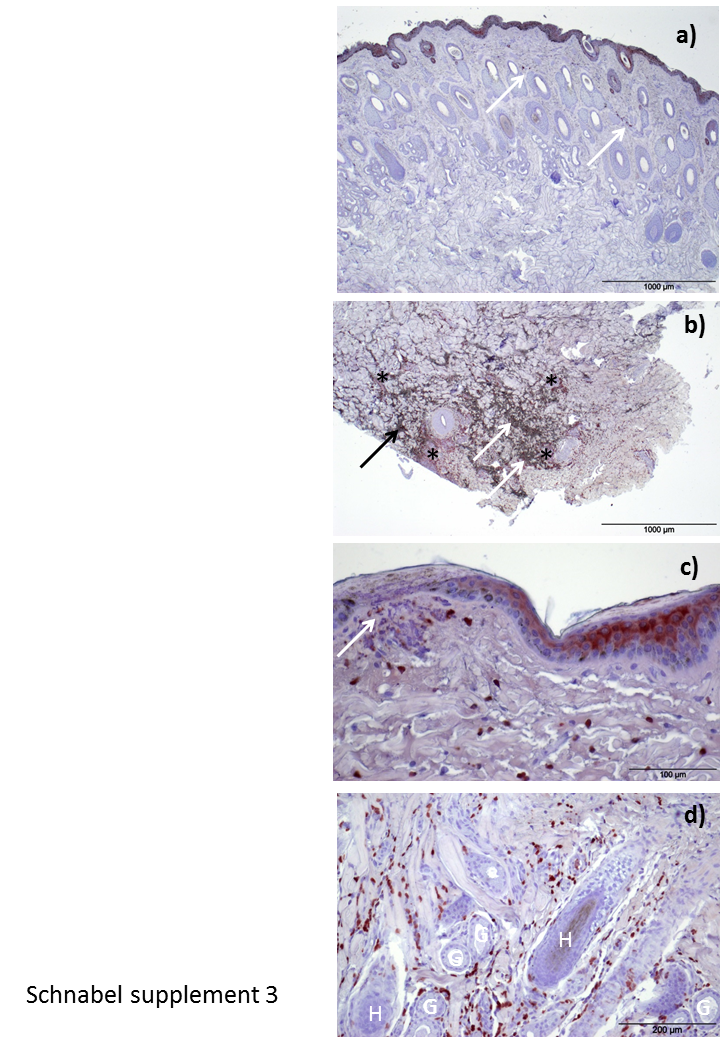

Supplement: Additional file 6: — Immunohistochemistry for calprotectin. Immunohistochemical localisation of calprotectin (red) in representative skin samples (counterstained with haematoxylin). a) Overview of Ep and Dermis with low grade inflammation. Calprotectin-positive cells are present in Dpap (arrows). In addition, Ep and hairs were immunopositive in parts. b) Overview of Dret with haemorrhage (arrows). Some leukocytes (asterisks) near and within haemorrhage stained positive for calprotectin (red). c) Epithelial Ulcer (arrow). Subepithelial leukocytes frequently stained calprotectin-positive (red). In addition, parts of the Ep (Str. spinosum and Str. granulosum) are positive for calprotectin. d) Dpap with adnexa. Infiltrating calprotectin-positive (red) leukocytes in perivascular localisation indicate a distinct inflammation. Hairs (H) and excretory ducts of apocrine glands (G) were negative. [file 12917_2015_452_MOESM6_ESM.png]

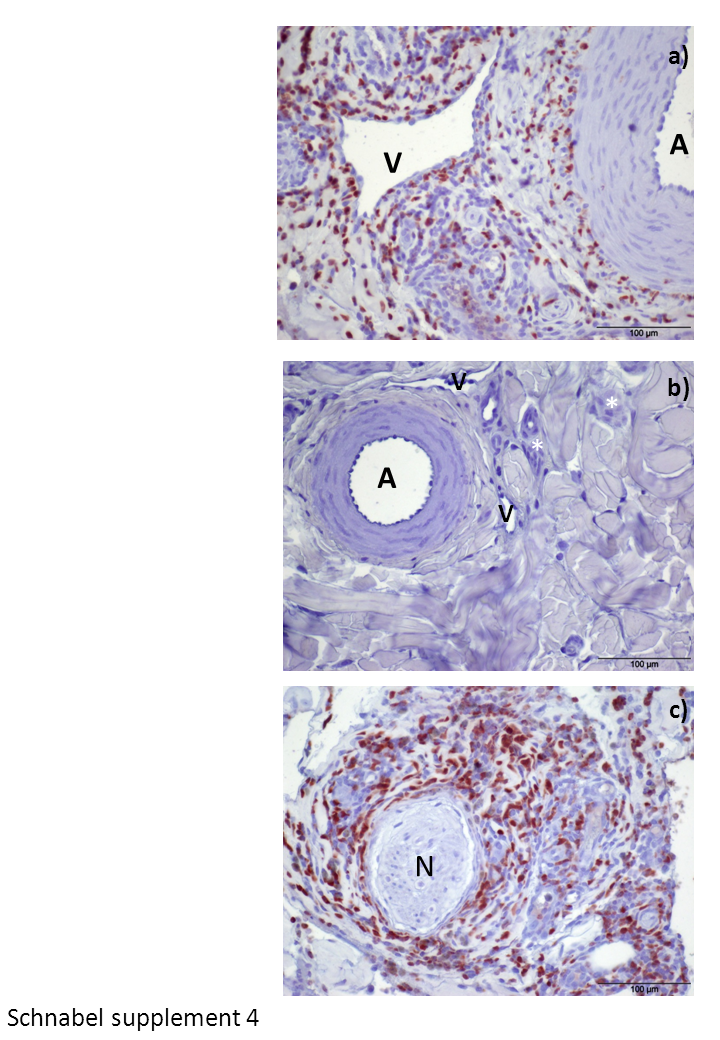

Supplement: Additional file 7: — Detail of calprotectin immunohistochemistry (continued). Immunohistochemical localisation of calprotectin (red) in representative skin samples (counterstained with haematoxylin), a) Dret with perivascular inflammation around larger vessels. Detail of large artery (A) and vein (V), which are surrounded by multiple calprotectin-positive (red) leukocytes. b) Dret – Isotype control. Similar localisation as a) with large artery (A) and smaller veins (V), where a perivascular infiltration (asterisks) is visible, but red staining is absent (negative). c) Perineural leukocyte infiltration in Dret. Detail of nerve (N) and vessels; note the distinct perivascular and perineural infiltration with calprotectin-positive leukocytes. [file 12917_2015_452_MOESM7_ESM.png]

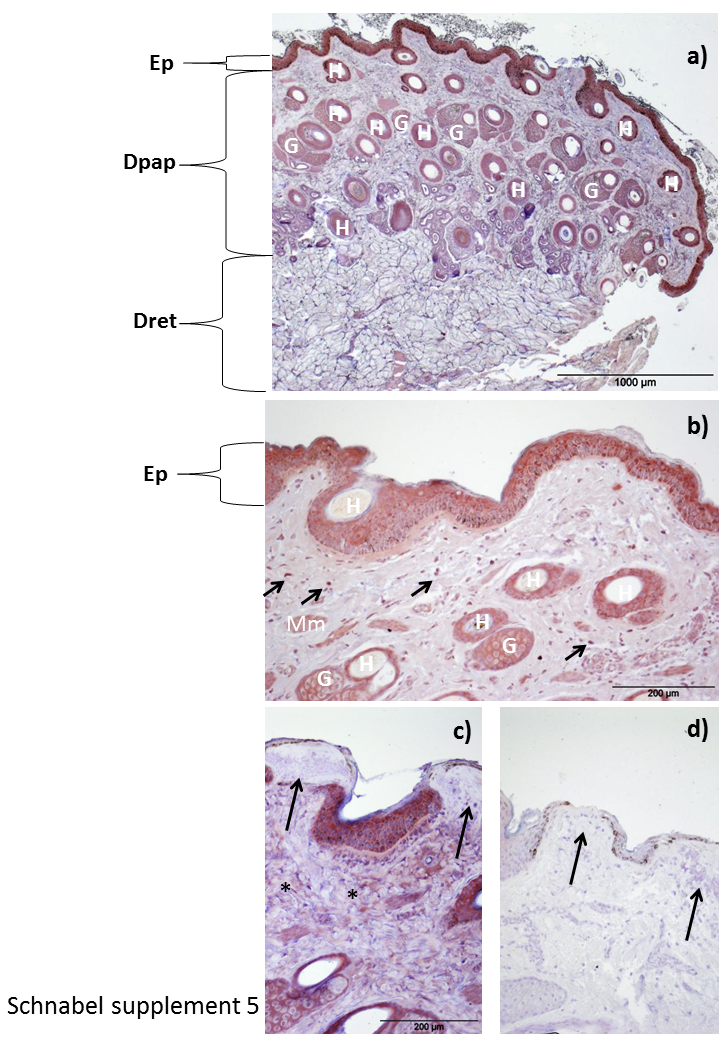

Supplement: Additional file 8: — Immunohistochemistry for Interleukin 12. Immunostaining for IL-12 (red) illustrated in representative skin samples (counterstaining with haematoxylin). a) Overview of Ep and dermis in inflamed skin. Ep, hairs (H) and sebaceous glands (G) stained immunopositive for IL-12 (red). b) Detail of Ep and Dpap. The strata granulosum, spinosum and basale of the stratified squamous epithelium (Ep) showed a cytoplasmic red staining for IL-12. Sebaceous glands (G) and hairs (H) usually stained positive for IL-12. Musculi arrectores pilorum (Mm) were also positive for IL-12. In addition, infiltrating leukocytes were frequently positive for IL-12 (arrows). c) Epithelial ulcers. Ulcers (arrows) were unstained in otherwise IL-12-positive stained squamous epithelium. Parts of the fibrocytes (asterisks) in Dpap also stained positive for IL-12. d) Isotype control. No immunopositive staining is visible in a similar localisation as in c). [file 12917_2015_452_MOESM8_ESM.png]

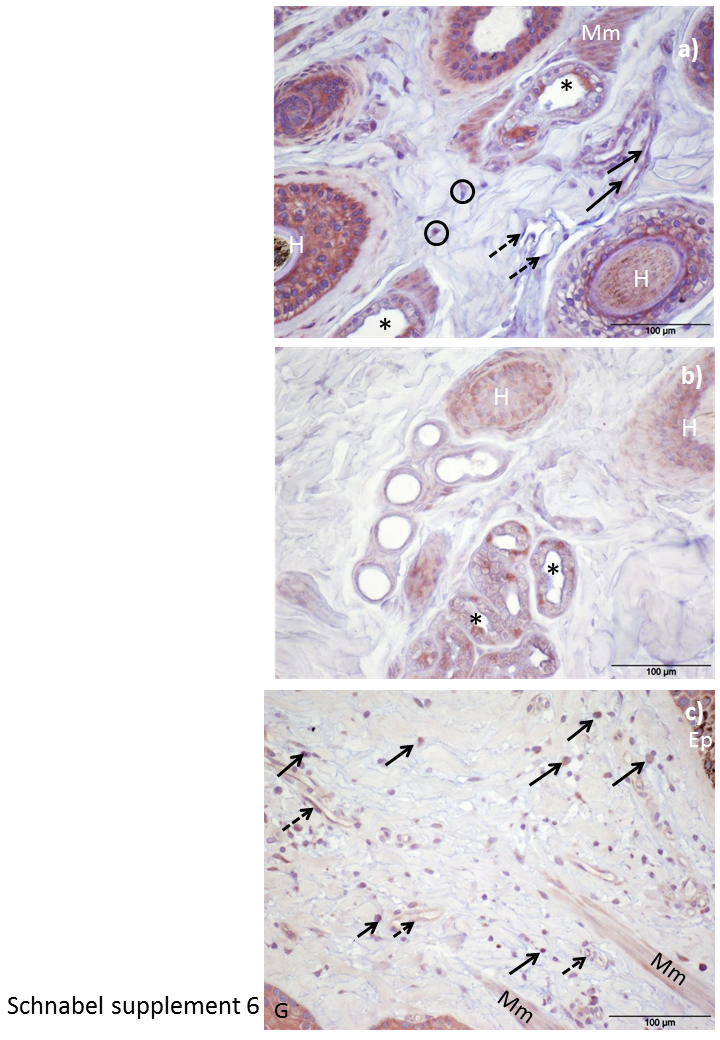

Supplement: Additional file 9: — Immunohistochemistry for Interleukin 12 in papillary dermis. Red immunoreactions for IL-12 are shown in representative skin samples with haematoxylin counterstaining. a) Detail of Dpap with adnexa. Epithelia of various kinds were usually positive for IL-12, e.g. cortex and epithelial root sheath of hairs (H), and glands and their excretory ducts (asterisks). Please note the patchy staining pattern of the excretory ducts. Endothelia stained positive (arrows) or negative (dashed arrows) for IL-12. Additionally, Mm. arrectores pilorum (Mm) stained positive for IL-12. Fibrocytes usually appeared negative for IL-12, but some perinuclear immunopositive staining of fusiform cells in the connective tissue could be noted (circles). b) Detail of Dpap illustrating appearance of apocrine glands. Apocrine glands were positive for IL-12 (asterisks), but showed a patchy staining. Epithelial components of hairs (H) were immunopositive, while fibrocytes in this detail were negative for IL-12.c) Dpap – detail of subepithelial inflammation. Infiltrating leukocytes can be found perivascularly and are dispersed diffusely in the connective tissue. They frequently show a red perinuclear and cytoplasmic immunostaining for IL-12 (arrows). Mm. arrectores pilorum (Mm) and endothelia (dashed arrows) stained positive for IL-12, while fibrocytes were usually negative for IL-12. Epithelium (Ep) and a gland (G) are indicated at the edges of the image (top right and bottom left, respectively). [file 12917_2015_452_MOESM9_ESM.png]

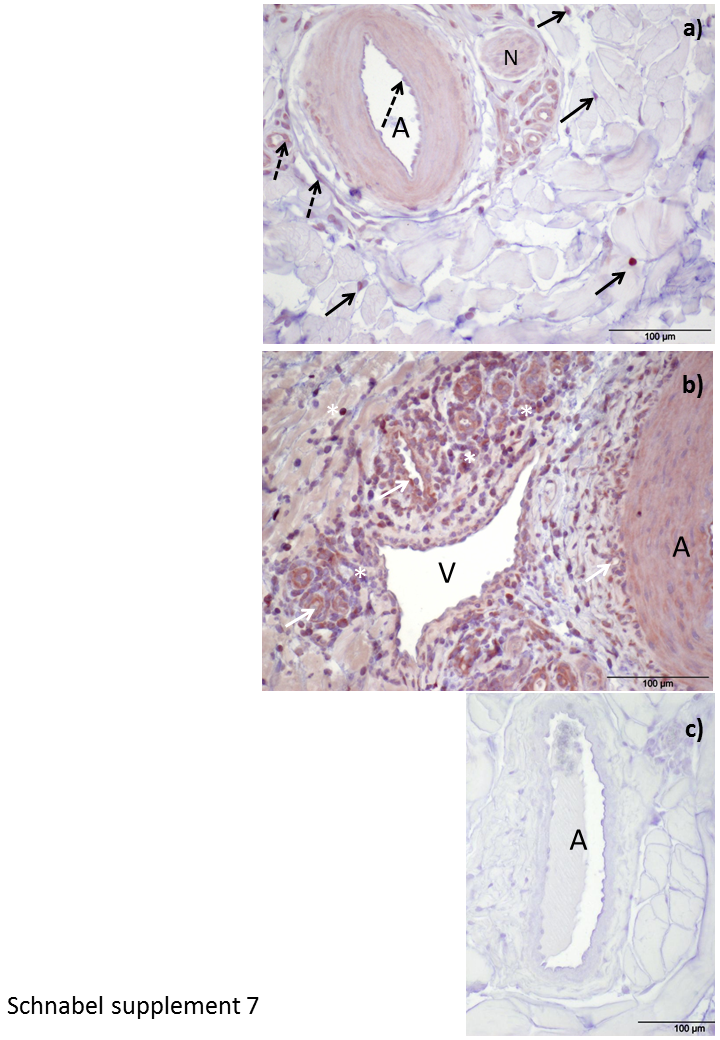

Supplement: Additional file 10: — Immunohistochemistry for Interleukin 12 in reticular dermis. Immunostaining for IL-12 (red) in representative skin samples (counterstaining with haematoxylin). a) Large artery and nerve in Dret. Walls and endothelia (dashed arrows) of a large artery (A) and small blood vessels stained positive for IL-12, as did the nerve (N). In addition, infiltrated leukocytes (arrows) were positive for IL-12. Fibrocytes and extracellular matrix were usually negative. b) Dret – detail with perivascular inflammation. Leukocytic perivascular infiltration of high degree proximate to artery (A) and vein (V). In addition, vessel walls and endothelia (arrows) infiltrated cells (asterisks) are positive for IL-12. Fibrocytes and extracellular matrix are negative apart from a weak background staining. c) Isotype control Dret with artery (A). All tissue components are negative in detail of Dret. [file 12917_2015_452_MOESM10_ESM.png]

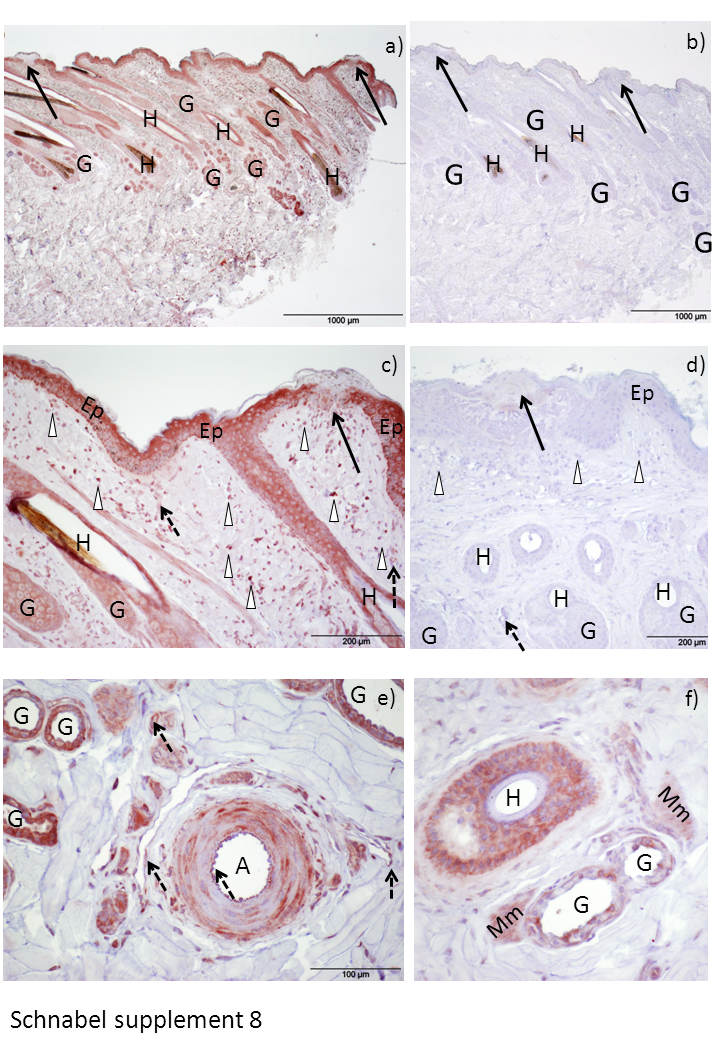

Supplement: Additional file 11: — Immunohistochemistry for Interleukin 18. Illustration of red IL-18 immunoreaction in representative skin samples (counterstained with haematoxylin). a) Overview of Ep and dermis in mildly inflamed skin. Ep (except for ulcers, arrows), hairs (H) and glands (G) stained positive for IL-18. b) Isotype control – overview of Ep and dermis. No immunopositive staining is visible in a similar localisation as in a) in the papillary layer. c) Detail of Ep with ulcer (arrow) and Dpap with medium grade leukocyte infiltration. All epithelial tissues [stratum granulosum, spinosum and basale of the stratified squamous epithelium (Ep), cortex and epithelial root sheath of hairs (arrowheads), sebaceous glands (G)] showed a cytoplasmic immunoreaction for IL-18, while ulcers (arrow) were negative for IL-18. Infiltrating leukocytes (arrowheads) were frequently positive for IL-18. The IL-18 staining of fibrocytes and endothelia (dashed arrows) varied from positive to negative. d) Detail of Ep and Dpap, isotype control. A corresponding section to c) did not show any reaction for IL-18. Symbols: stratified squamous epithelium (Ep) with an ulcer (arrow), hairs (cross-section, H), sebaceous glands (G), endothelia (dashed arrows) and leukocyte infiltration (arrowheads). e) Detail of Dpap with vessels and excretory ducts of apocrine glands (G). Blood vessel walls and endothelia (dashed arrows) of a larger artery (A) and smaller vessels stained positive for IL-18 (dashed arrows) usually appeared with immunopositive Lamina media and endothelia (dashed arrows). Excretory ducts of apocrine glands (G) were positive for IL-18. Fibrocytes and extracellular matrix were usually negative. f) Detail of adnexa in Dpap. The epithelial root sheath of a hair (H) and Mm. arrectores pilorum (Mm) always stained immunopositive for IL-18, while apocrine glands (G) stained positive, but with some patchy appearance. Fibrocytes and extracellular matrix were usually negative. [file 12917_2015_452_MOESM11_ESM.png]

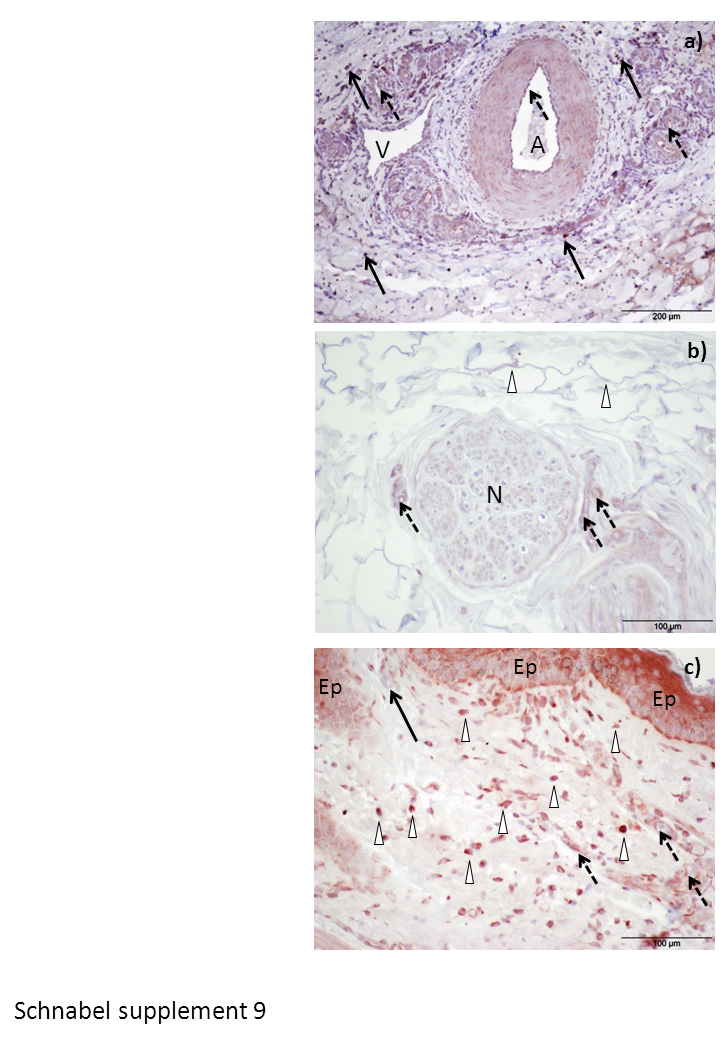

Supplement: Additional file 12: — Immunohistochemistry for Interleukin 18 (continued). Immunostaining for IL-18 (red) in representative skin samples (counterstaining with haematoxylin). a) Dret – detail with perivascular inflammation. Leukocytic perivascular infiltration is visible proximate to artery (A) and vein (V). Vessel walls and endothelia (dashed arrows), as well as infiltrated cells (arrows) are positive for IL-18. Fibrocytes and extracellular matrix are negative, apart from a weak background staining. b) Cross-sectioned nerve (N) in subcutis. Most parts of the nerve (N) stained immunopositive. In addition, adjacent capillary endothelia were positive for IL-18 (dashed arrows). c) Detail of infiltrated cells in Dpap. Infiltrating subepithelial immune cells were usually positive for IL-18 (arrowheads), as well as capillary endothelia (dashed arrows). Positive stratified squamous epithelium (Ep) and negative basis of an ulcer (arrow) are visible at the top edge of the detail. [file 12917_2015_452_MOESM12_ESM.png]

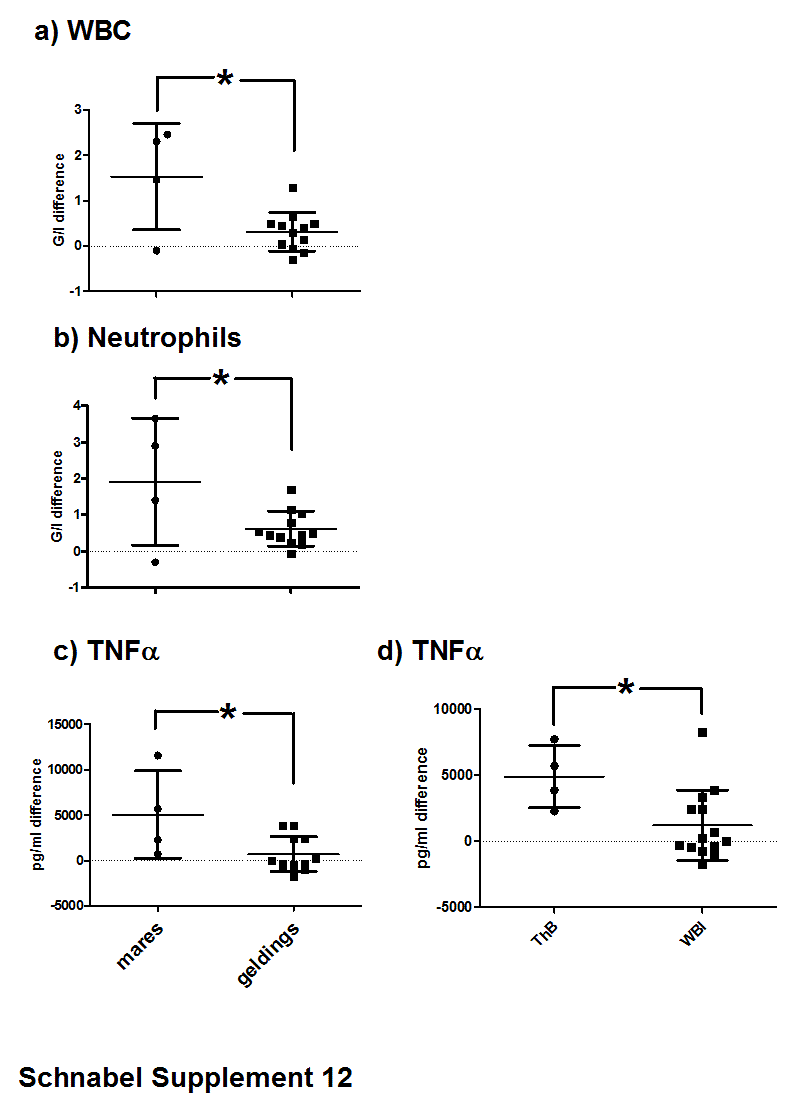

Supplement: Additional file 14: — Influence of sex and type on systemic parameters. Differences to individual baselines of a) WBC, b) neutrophils and c, d) TNFα release (in medium only) of horses treated with DNA (groups B–D) calculated at t12 and t24 are plotted in histograms for mares and geldings (a–c) or Thoroughbreds (ThB) and Warmbloods (WBl) (d). Horizontal bars represent mean and SD. Asterisks (*) with brackets (┌ ┐) represent significantly different comparisons. The WBC, neutrophil and TNFα increases to individual baselines after treatments with SAINT-18 formulated DNA were significantly higher in mares than in geldings. Increases of TNFα releases were significantly higher in ThB than in WBl. [file 12917_2015_452_MOESM14_ESM.png]
