# Supplementary material for: Immune response of healthy horses to DNA constructs formulated with a cationic lipid transfection reagent
Source: BMC Vet Res. 2015 Jun 23;11:140. doi: 10.1186/s12917-015-0452-3 (PMC4476236; doi:10.1186/s12917-015-0452-3)
Supplement: Additional file 5: — Evaluation of shaving and scrubbing effects on skin irritation. Description of an additional experiment to evaluate the impact of shaving and scrubbing on skin alterations. [file 12917_2015_452_MOESM5_ESM.docx]

Schnabel et al.

# Supplement 11 Evaluation of shaving and scrubbing effects on skin irritation

# Materials and methods:

Six additional clinically healthy horses (one mare, three geldings and two stallions) aged 3 to 24 years (mean 13.2 years) with a body weight of 520 kg to 663 kg (mean 579 kg) were included in the study. There was one ThB (English ThB) and five WBls (two Oldenburg WBls, two German WBls and one Standardbred). One horse was grey, four were brown and one was piebald.

These horses were euthanized in the course of an unrelated study on diagnostic imaging (animal experiment No. 33. 14-42502-04-13/1219) and were clipped and shaved 60 h and scrubbed 24 h before euthanasia, as described above for preparation of i.d. injections (groups A – D), at one site on the neck. Immediately postmortem, skin biopsy specimens were taken from this site (treatment E, shaving samples) and from another site which was only shaved and briefly scrubbed immediately before sampling (control, healthy skin).

Biopsy specimens were treated and analysed as described for the samples of groups A – D.

# Results

Macroscopically shaved and scrubbed skin was unchanged at the time of sampling.

Differences between treatment and control samples from the *Ep* were significantly different between treatments (Kruskal-Wallis test, p = 0.0049). Shaving and scrubbing resulted in ulceration of the *Ep*. Significantly higher grades (scores) of ulceration could be found in comparison to untreated controls (Wilcoxon signed-rank test, p = 0.0313), which showed no ulcers at all.

An increase of macrophages in the *Dpap* in a diffuse pattern was noticed after shaving only in comparison to untreated samples (p = 0.0625).

Higher scores in the *Dpap* and *Dret* were found for treatment and control sites in groups A – D than in shaved horses, without reaching statistical significance. There were more neutrophils in the dermis (*Dpap* and *Dret*) of samples of A – D (independent of *ctrl* or *treat* sites) than in the samples of shaved horses (E).

# Discussion and conclusion

Shaving and scrubbing alone induce ulcerative dermatitis. Effects in the dermis are usually limited to superficial layers. Treatment effects of i.d. injections in A – D are not likely to be hidden by shaving effects.

Shaving, if necessary, should be performed immediately prior to sampling to prevent iatrogenic ulcerative dermatitis and artefacts in samples.
