# Supplementary material for: Immune response of healthy horses to DNA constructs formulated with a cationic lipid transfection reagent
Source: BMC Vet Res. 2015 Jun 23;11:140. doi: 10.1186/s12917-015-0452-3 (PMC4476236; doi:10.1186/s12917-015-0452-3)
Supplement: Additional file 13: — Staining characteristics of IL-12 and IL-18 in IHC. Detailed description of IL-12 and IL-18 stainings of skin biopsies. [file 12917_2015_452_MOESM13_ESM.docx]

# Schnabel et al.

# Supplement 10

# Staining characteristics of IL-12 and IL-18

# As the results of IHC stainings for these two cytokines were very similar, the description of these will be presented together.

## Stained tissues and cell types

# The *strata granulosum, spinosum* and *basale* of the stratified squamous epithelium always stained cytoplasmically positive for IL-12 and IL-18*.* Melanocytes were difficult to evaluate due to the intensive dark brown colour of the melanin granules contained. Hairs were positive for IL-12 and IL-18 in the cortex and epithelial root sheath. Sebaceous glands were also positive for both cytokines, except for necrotic areas. Apocrine glands and their excretory ducts usually stained positive for IL-12 and IL-18 with some patchy appearances and a few exceptions of unstained glands. Myoepithelial cells around sweat glands always stained positive. Muscles (skeletal and smooth) were also always weakly immunostained (see Supplements 5a-c; 6a, b; 8a, c, e, f; 9c).

# Fibrocytes in the connective tissue layers of the skin were either stained or unstained for IL-12 and IL-18; however, when stained, the immunoreaction was found in the cytoplasm. More positive fibrocytes were found after treatments than in healthy non-treated skin. Nevertheless, fibres and extracellular matrix were usually negative, but the appearance varied due to some background staining in more severely inflamed tissues. Plasma in vessels and intercellular fluid (oedema or exudate) was often found to be weakly stained (see Supplements 5a-c; 7a; 8a, c, e, f; 9a, c).

# Adipocytes were found to be negative or positive for IL-12 and IL-18. Positive cells were usually associated with adjacent inflammation and there was often some uncertainty about the differentiation of adipocytes from inflammatory cells (data not shown).

# Endothelia were usually positive for IL-12 and IL-18 with a few exceptions. Negative endothelia were found more often in control tissues of healthy non-treated equine skin, but hardly at all in any capillaries. Pericytes of capillaries were usually positive for IL-12 and IL-18. Vessel walls were generally positive for IL-12 and IL-18 in the *Tunica media* (see Supplements 6c; 7a, b; 8e, f; 9a, c). Nerves were positive for IL-12 and IL-18 in parts (see Supplements 7b; 9b).

# Infiltrating immune cells were frequently positive for IL-12 and IL-18. A specifically strong immunoreaction was observed in macrophages and some lymphocytes (see Supplements 5b; 6a, c; 7c, d; 8c, e, f; 9a, c;).

# Staining intensities varied between and within individual samples, especially in immune cells.

# Isotype controls showed no staining at all, except for very pale background staining in one necrotic area of an epithelial ulcer (see Supplements 5d; 7c; 8b, d).
